# Enhanced in vitro immersion behavior and antibacterial activity of NiTi orthopedic biomaterial by HAp-Nb_2_O_5_ composite deposits

**DOI:** 10.1038/s41598-023-43393-3

**Published:** 2023-09-25

**Authors:** Mir Saman Safavi, Jafar Khalil-Allafi, Elisa Restivo, Arash Ghalandarzadeh, Milad Hosseini, Giacomo Dacarro, Lorenzo Malavasi, Antonella Milella, Andrea Listorti, Livia Visai

**Affiliations:** 1 Research Center for Advanced Materials, Faculty of Materials Engineering, Sahand University of Technology, P.O. Box: 51335-1996, Tabriz, Iran; 2 Molecular Medicine Department (DMM), Center for Health Technologies (CHT), UdR INSTM, University of Pavia, Viale Taramelli 3/B, 27100 Pavia, Italy; 3 Medicina Clinica-Specialistica, UOR5 Laboratorio di Nanotecnologie, ICS Maugeri, IRCCS, 27100 Pavia, Italy; 4 School of Metallurgy and Materials Engineering, Iran University of Science and Technology, Tehran, Iran; 5 Department of Chemistry, Physical Chemistry section, and CHT, University of Pavia, Viale Taramelli 12, 27100 Pavia, Italy; 6 Department of Chemistry and INSTM, University of Pavia, Viale Taramelli 12, 27100 Pavia, Italy; 7 Department of Chemistry, University of Bari Aldo Moro, Via Orabona 4, 70125 Bari, Italy

**Keywords:** Microbiology, Chemistry, Materials science

## Abstract

NiTi is a class of metallic biomaterials, benefit from superelastic behavior, high biocompatibility, and favorable mechanical properties close to that of bone. However, the Ni ion leaching, poor bioactivity, and antibacterial activity limit its clinical applications. In this study, HAp-Nb_2_O_5_ composite layers were PC electrodeposited from aqueous electrolytes containing different concentrations of the Nb_2_O_5_ particles, i.e., 0–1 g/L, to evaluate the influence of the applied surface engineering strategy on in vitro immersion behavior, Ni^2+^ ion leaching level, and antibacterial activity of the bare NiTi. Surface characteristics of the electrodeposited layers were analyzed using SEM, TEM, XPS, and AFM. The immersion behavior of the samples was comprehensively investigated through SBF and long-term PBS soaking. Gram-negative *Escherichia coli* (*E. coli*) and Gram-positive *Staphylococcus aureus* (*S. aureus*) infective reference bacteria were employed to address the antibacterial activity of the samples. The results illustrated that the included particles led to more compact and smoother layers. Unlike bare NiTi, composite layers stimulated apatite formation upon immersion in both SBF and PBS media. The concentration of the released Ni^2+^ ion from the composite layer, containing 0.50 g/L Nb_2_O_5_ was ≈ 60% less than that of bare NiTi within 30 days of immersion in the corrosive PBS solution. The Nb_2_O_5_-reinforced layers exhibited high anti-adhesive activity against both types of pathogenic bacteria. The hybrid metallic-ceramic system comprising HAp-Nb_2_O_5_-coated NiTi offers the prospect of a potential solution for clinical challenges facing the orthopedic application of NiTi.

## Introduction

Metallic biomaterials, including NiTi, Ti alloys, and stainless steels, have garnered a rising interest in a variety of clinical applications so that they hold a dominant position in the global implant market^1–7^. NiTi is a class of shape memory alloys that derive its name from the ability that allows it to restore the original shape even after deformation, by heating. Besides, the beneficial properties of the NiTi, such as high biocompatibility, superelastic behavior, suitable mechanical properties close to that of bone, and moderate corrosion resistance, make it an encouraging candidate for orthopedic, orthodontic, and cardiovascular applications, but the Ni ion leaching, poor bioactivity, and insufficient antibacterial activity still bring technical constraints and limit its practiced applications due to the increased possibility of implantation failure^8–13^.

To date, a variety of strategies, such as employing efficient fabrication methods, controlling fabrication parameters, the addition of favorable dopants during the fabrication of the bulk implant, and the application of fruitful surface modification technologies, have been proposed to overcome these drawbacks^14–18^. The surface finishing of NiTi implant with CaP family, in particular HAp, using the simple, cheap, and one-pot electrodeposition technology not only addresses the majority of the facing challenges but also converts them into opportunities^19^. A feasible approach to simultaneously promote the useful properties of HAp coatings and meet its common drawbacks, including poor antibacterial activity and mechano-corrosion behavior, is the introduction of a favorable second phase to the microstructure of the growing deposit. The post-implantation infections are of prime importance since they put additional health and economic burden on the patient. Thus, it is better to use a reinforcing phase that can enhance the antibacterial performance of the pure HAp even if the obtained improvement is not too significant^20–23^.

The tremendous features of Nb_2_O_5_, such as excellent thermal and chemical stability, extraordinary resistance to both wear and corrosion, outstanding bioactivity, and prominent biocompatibility, have triggered the wide spectrum of R & D activities to answer the question if it is possible to exploit Nb_2_O_5_ in biomedical applications. The reported results have confirmed the positive contribution of the Nb_2_O_5_ phase to the fabrication of the efficient biomedical coatings^24–26^.

In light of the abovementioned statements, and considering the increasing demand for efficient biomaterials, the use of Nb_2_O_5_ particles as the reinforcing phase to bypass the deficiencies associated with the pure HAp electrodeposits seems reasonable. This survey endeavors to enhance the surface characteristics, in vitro bioactivity, and antibacterial activity of HAp coatings reinforced by the Nb_2_O_5_ particles to provide a superior surface modification of the synthetic NiTi implant. Although the Nb_2_O_5_ particles are not famous for their antibacterial performance, the results of this contribution are believed to shed new light on developing the next generation of high-performance Nb_2_O_5_-containing coatings.

## Materials and methods

### Substrate preparation and electrodeposition

The protocol applied for the pretreatment of the NiTi substrates and the employed electroplating parameters have been addressed in previous work^27^. Table 1 outlines the chemicals used for the preparation of the electrolyte and the operating parameters employed for the electrodeposition of the layers.

**Table 1:** The chemicals used for the preparation of the electrolyte and the employed operating parameters for the electrodeposition of the layers.

Label	Ca(NO_3_)_2_⋅4H_2_O (M)	NH_4_H_2_PO_4_ (M)	H_2_O_2_ (mL/L)	Nb_2_O_5_ (g/L)
HANb0	0.042	0.025	6	–
HANb1	0.042	0.025	6	0.25
HANb2	0.042	0.025	6	0.50
HANb3	0.042	0.025	6	1.0

Operating parameters	Amount			
Anode (exposed area)	Graphite plate (7 cm^2^)			
Cathode (exposed area)	NiTi disk (1 cm^2^)			
Interelectrode gap	3 cm			
Electrolyte temperature	70 °C			
Electrolyte pH	4.3			
Stirring speed	200 rpm			
Current density	15 mA/cm^2^			
Duty cycle	10%			
Plating time	20 min			

### Characterization of the studied samples

#### Surface morphology

The surface morphology of the as-deposited HAp-based layers was examined via a Zeiss EVO-MA10 SEM (Carl Zeiss, Oberkochen, Germany). For TEM observations (Jeol JEM-1200EXII, Japan), the electrodeposited layers were scratched from the NiTi substrate and dispersed in water using a conventional ultrasonic bath for 5 min. 10 µL of suspension was dropped on an amorphous carbon coated-300 mesh copper grid and allowed to dry out under a laboratory hood at room temperature for 24 h.

#### Chemical composition

The chemical composition of the surface was investigated by XPS analyses with a PHI 5000 Versa Probe II spectrometer (Physical Electronics) equipped with a monochromatic Al Kα X-ray source (1486.6 eV), operated at 15 kV and 24.8 W, with a spot size of 100 µm. Survey (0–1200 eV) and high-resolution spectra (C1s, O1s, Ca2p, P2p, and Nb 3d) were recorded in FAT mode at pass energy of 187.85 and 29.35 eV, respectively. Surface charging was compensated using a dual beam charge neutralization system. All spectra were collected at an angle of 45° with respect to the sample surface. The hydrocarbon component of C1s spectrum was used as an internal standard for charging correction and it was fixed at 284.8 eV. Spectra were processed with MultiPak software (Physical Electronics). Atomic concentrations were determined from the high-resolution spectra after subtracting a Shirley-type background, using the Scofield sensitivity factors set in the MultiPak software.

#### Surface topography

The surface roughness of the layers was determined by AFM (Nanosurf Mobile S, Switzerland).

#### Surface wettability

The wettability of the various surfaces was measured by the sessile drop method employing a static/dynamic contact angle measurement instrument (KSV CAM200, KSV Instruments, Finland) at 25 ± 1 °C.

#### In vitro immersion behavior

The in vitro immersion behavior of the samples was determined in the SBF and PBS media.

#### SBF

The protocol applied for assessing the in vitro immersion behavior of the sterilized bare and coated samples was comprehensively addressed in our previously published paper^27^.

#### PBS

The samples were UV-sterilized for 30 min, followed by autoclaving for 20 min at 121 °C before immersion in PBS. The bare and coated specimens were incubated in 10 ml of PBS at 37 ± 0.5 °C. The solution was collected and changed with a fresh one at specified intervals, i.e., 1, 3, 7, 14, and 30 days. Then, 100 µL of HNO_3_ was added to the collected media, and the resultant media were kept in the freezer at − 32 °C. All of the experimental procedures were carried out under sterile conditions.

The surface morphology and elemental constituents of the SBF- and PBS-immersed specimens were studied by a FESEM (MIRA3 TESCAN, Czech Republic) equipped with EDS. The phase composition of the PBS-soaked samples was determined by XRD Cu-Kα radiation (Bruker D8 Advance, Germany), working at voltage and current of 40 kV and 40 mA, respectively. The spectra were collected in the 2Ө range from 10 to 60° with a step size of 0.04°. The concentration of released Ni^2+^ and Ca^2+^ ions in the PBS solution upon soaking at specified intervals was measured via ICP-MS (Elan DRC-e, Perkin Elmer, Shelton, USA) and ICP-OES (OPTIMA 3300 DV Perkin Elmer, Shelton, USA), respectively.

### Bacterial strains and culture conditions

The microorganisms used were a Gram-negative strain, *Escherichia coli* ATCC 25922 (*E. coli*), and a Gram-positive strain, *Staphylococcus aureus* ATCC 25923 (*S. aureus*), kindly obtained from the laboratory of Prof. R. Migliavacca (Department of Clinical-Surgical Diagnostic and Pediatric Sciences, Unit of Microbiology and Clinical Microbiology, University of Pavia, Italy). Bacteria were grown in 10 ml of appropriate medium overnight under aerobic conditions at 37 °C using a shaker incubator (VDRL Stirrer 711/CT, Asal Srl, Italy). *E. coli* was inoculated in Luria Bertani broth (LB) (ForMedium™, UK) whereas *S. aureus* was in TSB (Tryptic Soy Broth) (ForMedium™, UK). The number of bacterial cells/ml of both cultures was determined by comparing the optical density (OD600) of the sample with a standard curve relating OD600 to cell number^28–31^.

### Antibacterial assays

Before performing viability assays, samples were sterilized with 96% ethanol for 10 min, followed by UV light irradiation for 30 min, and eventually autoclaved for 20 min at 121 °C under a pressure of 1 bar. The antibacterial activity of the samples was first evaluated with the plate-counting, to screen the samples with antibacterial properties. Then, the antibacterial efficacy of the samples’ surface was investigated through an MTT assay.

#### Plate-counting assay

The bare and coated NiTi samples were placed in a tube, and *E. coli* and *S. aureus* bacteria at the concentration of 7.5 × 10^3^ CFU/ml were seeded on their surface. The tube was incubated under rotation for 3 h at 37 °C. Subsequently, 100 µL of the diluted suspension in 0.9 wt% NaCl was evenly spread on the nutrient agar plates, and the plates were incubated at 37 °C for 24 h. Finally, the formed colonies on the plates were counted. All the antibacterial experiments were performed in triplicate.

#### Evaluation of antibacterial activity by MTT

Sterile samples were washed twice in ddH_2_O and incubated with planktonic bacteria for 24 h at 37 °C. 2.5 × 10^3^ bacteria/sample were seeded on NiTi, HANb0, and HANb2 samples, where NiTi considered as control. The viability was assessed on planktonic bacteria remaining adherent on the samples' surface. The assay was performed through the quantitative MTT colorimetric assay (Sigma-Aldrich, St. Louis, SM, United States). This test measures dehydrogenase activity as an indicator of the bacterial metabolic state. MTT solution (5 mg/ml), dissolved in sterile PBS (0.134 M NaCl, 20 mM Na_2_HPO_4_, 20 mM NaH_2_PO_4_), was used as a stock solution at the working concentration of 0.5 mg/ml. The test was performed for 3 h at 37 °C. Upon the presence of viable bacteria, reduction of the MTT salt results in purple insoluble formazan granules that are dissolved in acidified 2-propanol (0.04 N HCl). The colorimetric reaction was analyzed at CLARIOstar (BMG Labtech, Ortenberg, Germany) at 570 nm wavelength with 630 nm as the reference wavelength. Titration curve interpolation was used to express the number of bacteria. Results were normalized to bacterial cells seeded on the bare NiTi. All the viability experiments were carried out in triplicate and repeated two times^31^.

### Statistical analysis of microbiological tests

All the statistical calculations were carried out by considering the mean of the results (in triplicate) obtained from two separate experiments. The analysis was carried out using GraphPad Prism 9 (GraphPad Inc, San Diego, CA, United States). Statistical analysis was performed using Student’s unpaired, two-sided t-test (significance level of p < 0.05) in comparison to NiTi control. In addition, a two-way analysis of variance (ANOVA), followed by Bonferroni’s multiple comparisons test was performed.

## Results and discussion

### Surface-related characteristics

**Figure 1:**
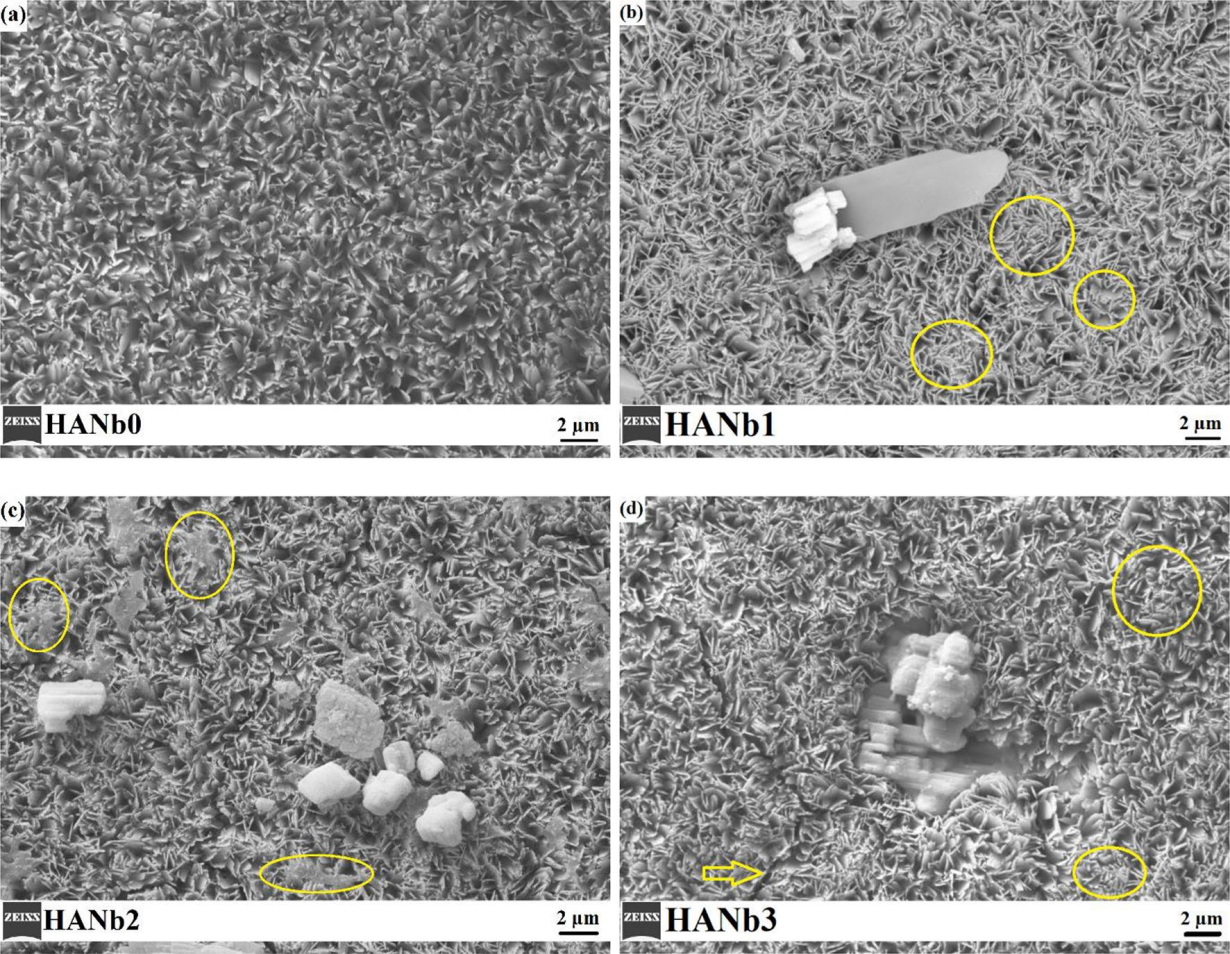
The surface morphology of the electroplated coatings: (**a**) HANb0, (**b**) HANb1, (**c**) HANb2, and (**d**) HANb3.

The surface morphology of the electroplated coatings is illustrated in Fig. 1. All of the coatings showed porous plate-like morphology; however, the thickness of the plates and the pore size changed with the co-deposition of the Nb_2_O_5_ particles. The vast majority of the constituent crystals were grown on faces perpendicular to the substrate. The HANb0 coating had the highest porosity content with the largest pores (see Fig. 1a)^32^. Moreover, there are several needle-like crystals throughout the microstructure of the HANb0 film. Please see the Supplementary Fig. I for the high-magnification SEM micrograph of HANb0 film, in which the needle-like crystals are clearly shown.

The inclusion of the particles led to the fusion and connection of the plate-like crystals, which are highlighted by circles in Fig. 1b–d. This can be attributed to the change in the growth direction of the HAp crystals with the inclusion of the Nb_2_O_5_ particles, as it has been illustrated in our previous paper^27^. Furthermore, the Nb_2_O_5_ particles placed on the surface may cover the pores and bridge the HAp crystals. The micron-sized plate in Fig. 1b assigns to the DCPD phase, which was precipitated along with HAp in the microstructure of the Nb_2_O_5_-containing composite layers. The microstructure of the HANb2 film is characterized by the lowest porosity content as well as the highest amount of fusion between the plate-like crystals (see Fig. 1c). The formation of the agglomerated particles as well as the nano-thick cracks, shown by the arrow in the microstructure of the HANb3 layer, confirms that the higher concentration of the Nb_2_O_5_ particles can adversely affect the morphological features of the HAp electrodeposits (see Fig. 1d).

**Figure 2:**
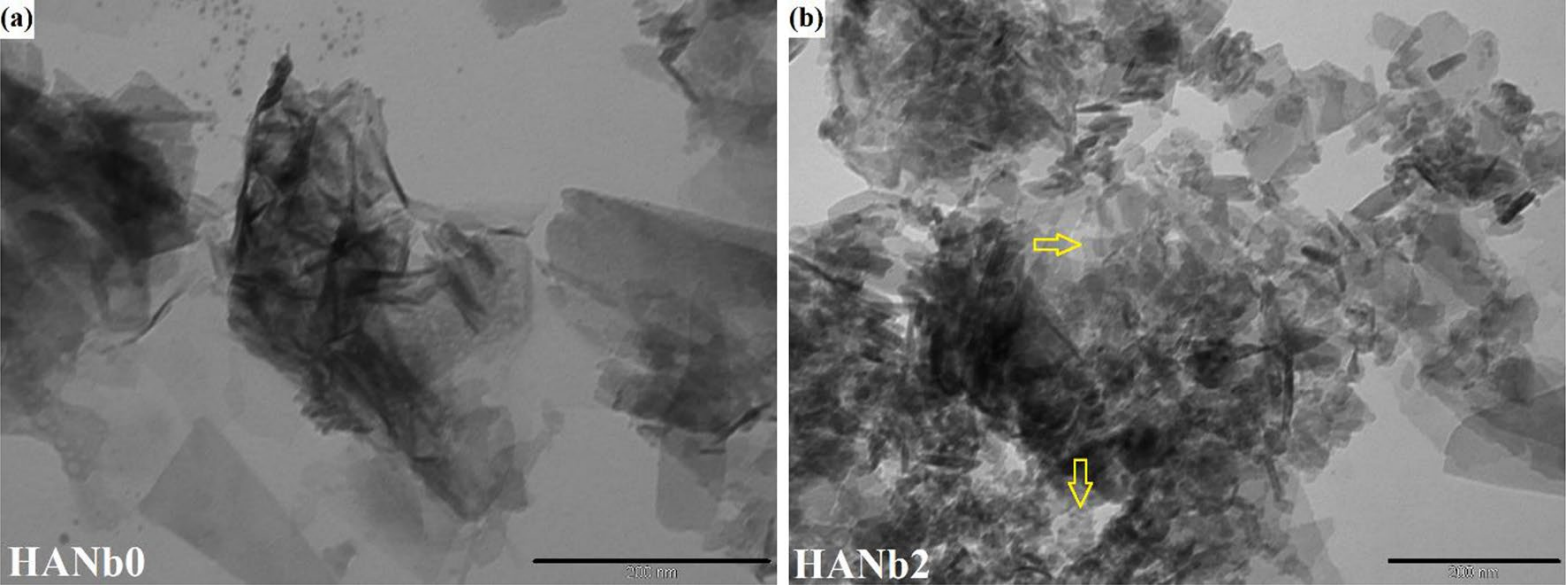
Bright-field TEM micrographs of (**a**) HANb0 and (**b**) HANb2 layers. Scale bars are 200 nm.

Figure 2 indicates the bright-field TEM micrographs of the electrodeposited layers. Figure 2a shows the nano-thick plates, which were grown along the c-axis perpendicular to the NiTi substrate. On the other hand, there are thicker plates, which encompass the Nb_2_O_5_ particles_,_ in the microstructure of the composite films (see Fig. 2b).

**Figure 3:**
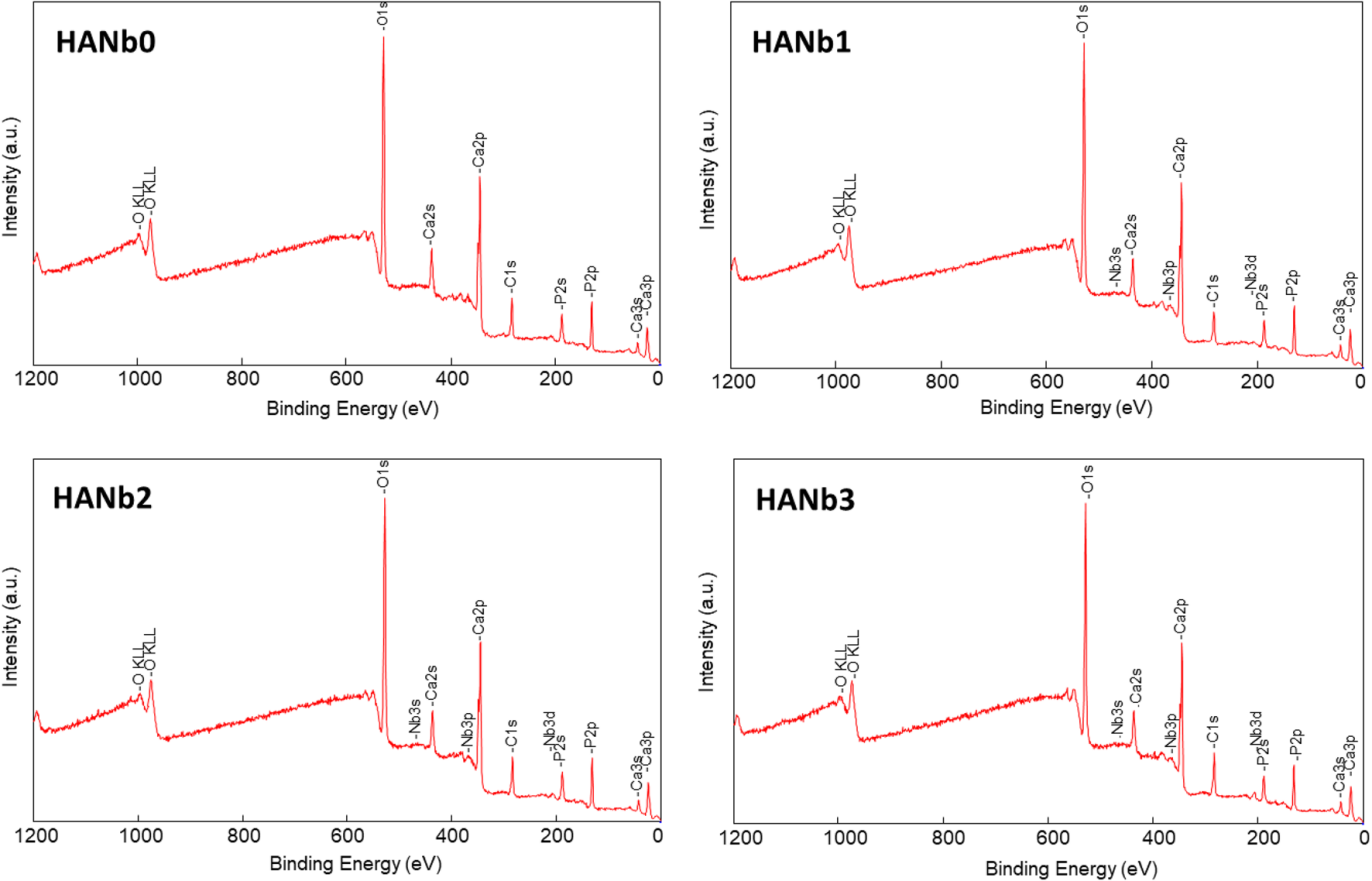
XPS survey spectra of outermost layer of the electrodeposited coatings.

XPS survey spectra of the outermost layer of the electroplated coatings are illustrated in Fig. 3. Besides Ca, P, and O, which are the main constituent elements of the HAp structure, there is a noticeable amount of C in the spectra of the samples due to the dissolving the present CO_2_ in the atmosphere in the aqueous electrolyte, in accordance with the literature^33^. The C 1s spectrum is fitted with four components peaked at 284.8 eV (C–C, C–H), 286.2 ± 0.1 eV (C–OH, C–OC), 287.9 ± 0.2 eV (C=O), and 289.0 eV ± 0.1 eV (COOH, CO_3_^2−^). For composite coatings, the additional Nb 3d spectrum consists of a doublet with the main component Nb 3d_5/2_ at 206.7 ± 0.2 eV.

**Figure 4:**
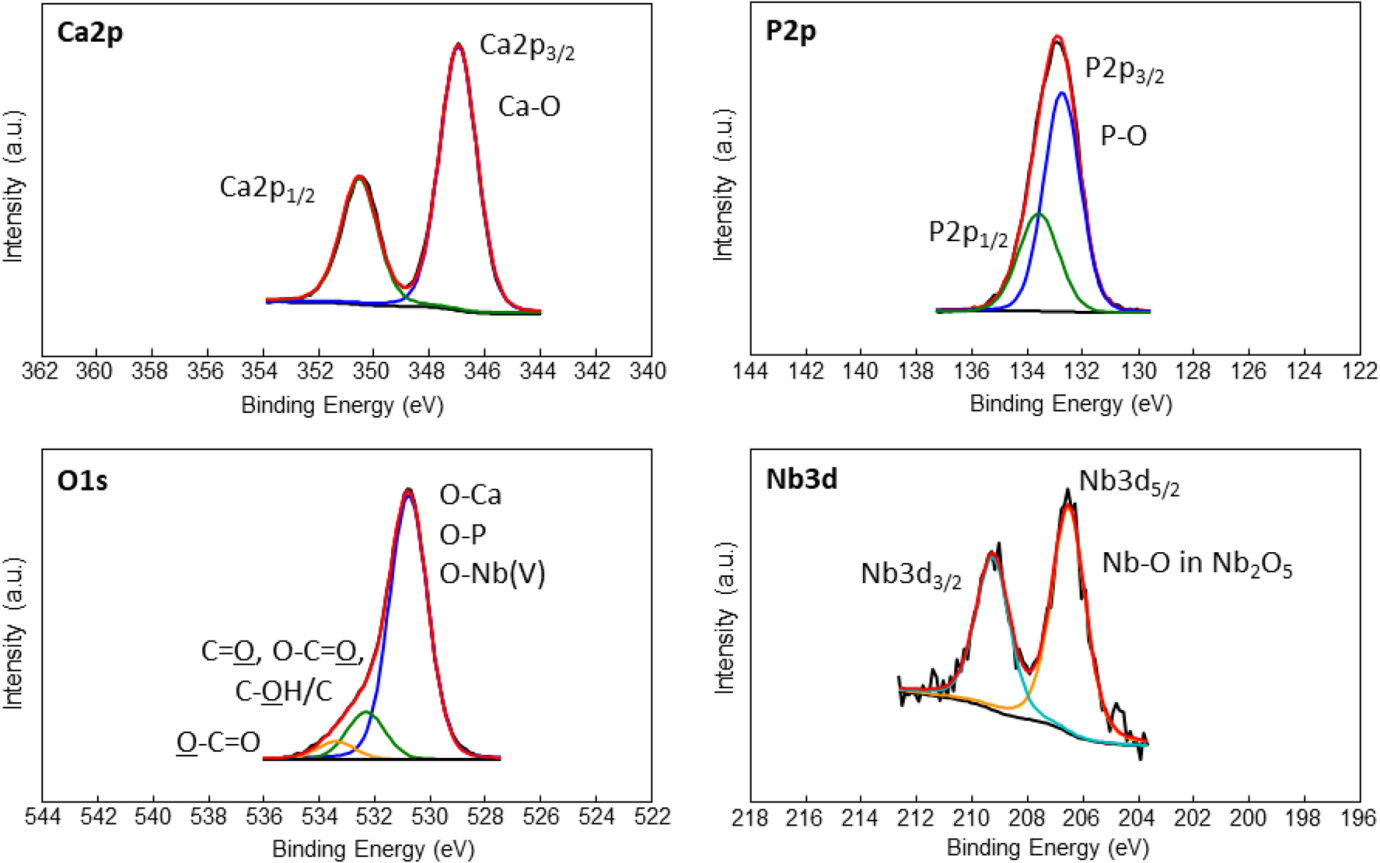
Representative high-resolution XPS spectra of Ca 2p, P 2p, O 1s, and Nb 3d regions for sample HANb3.

The high-resolution XPS spectra of Ca 2p, P 2p, O 1s, and Nb 3d regions for sample HANb3 are presented in Fig. 4. As it is seen, the binding energy values for Ca 2p_3/2_ and P 2p_3/2_ are 346.9 eV and 132.9 eV, respectively, which agree with the energies of Ca–O and P–O bonds in the microstructure of the standard HAp^34^. Furthermore, the O 1s peak at a binding energy of 531 eV corresponds to the hydroxyl ions of the HAp and oxide species^33^.

Table 2 outlines the binding energy values of the XPS peaks and those reported for the standard synthetic HAp^35^.

**Table 2:** Binding energy values of the XPS peaks and those reported for the standard synthetic HAp.

Materials	O 1s (eV)	Ca 2p_3/2_ (eV)	P 2p_3/2_ (eV)	Nb 3d_5/2_ (eV)
Standard HAp	531.5	347.5	133.5	–
HANb0	530.7	346.9	132.7	–
HANb1	530.8	347.0	132.9	207.0
HANb2	530.7	346.9	132.7	206.6
HANb3	530.7	346.9	132.7	206.5

The Nb 3d peak at a binding energy value of ≈ 207 eV and O 1 s peak at a binding energy of ≈ 530 eV approve the presence of the Nb_2_O_5_^36^. The Ca/P molar ratio of the coatings varies in the range of 1.25 ± 0.03 to 1.35 ± 0.05, while the Ca/P ratio of the natural bone obtained by XPS is 1.42 ± 0.02^37^. The content of the included Nb_2_O_5_ particles in the composite coatings is in the range of 0.1–0.3 at.%, and directly depends on their level in the electrolyte. Overall, the XPS results confirm the formation of the HAp phase and the successful inclusion of the Nb_2_O_5_ particles. To further highlight the differences in the chemical composition of the coatings, a comparison between the high-resolution spectra of the samples is made and the results are presented in Supplementary Fig. II. There is no difference detected neither in the peak shape nor in their binding energy position, however, for Nb 3d in which a slight difference of 0.5 eV was registered among the different samples (see Table 2). The mentioned difference is below the nominal energy resolution in the set conditions, i.e., 0.7 eV. The lack of differences in our opinion is related to the low amount of Nb included in the samples, which do not noticeably affect the structure of the material.

**Figure 5:**
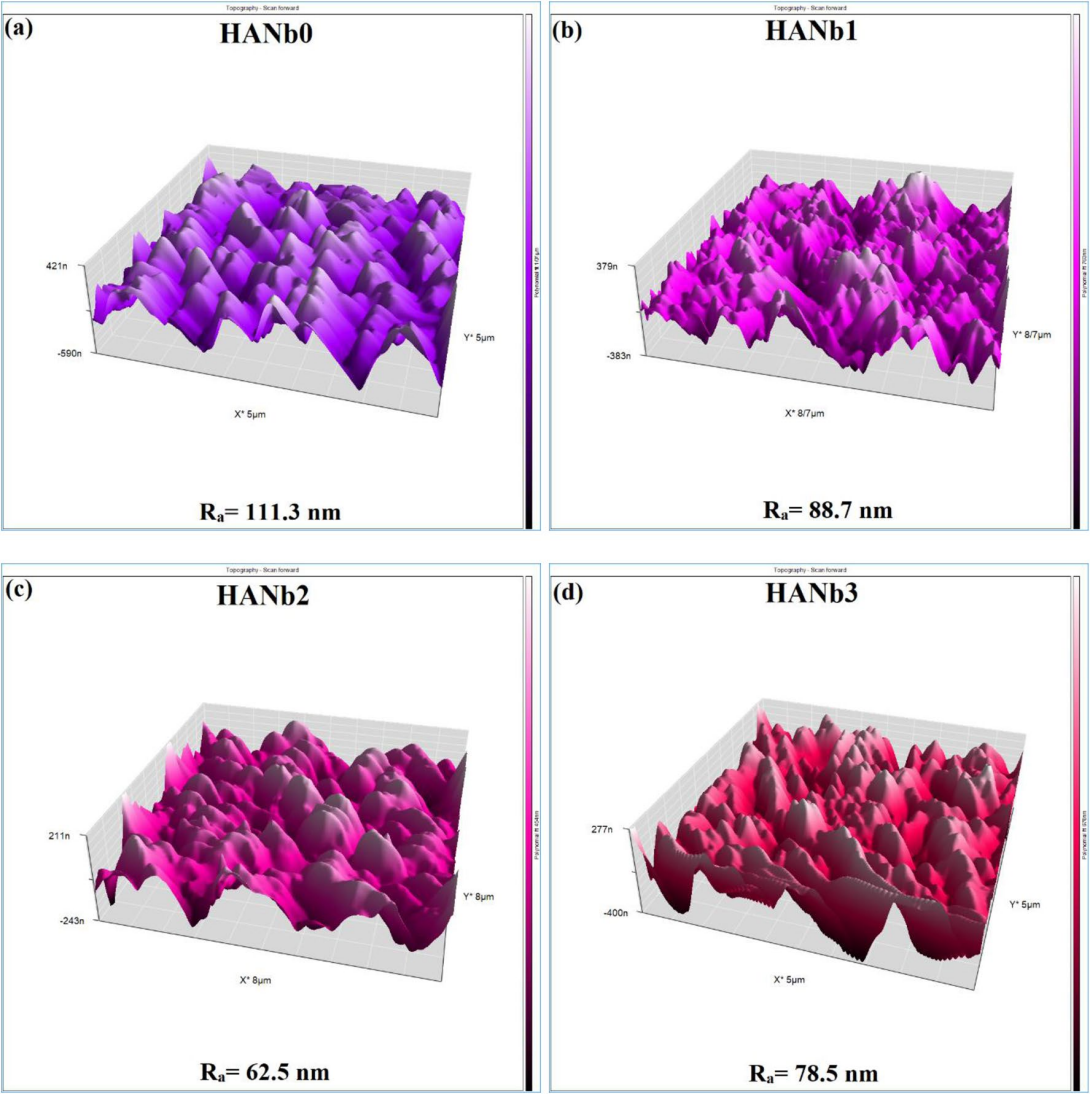
3D topographic AFM micrographs of the electrodeposits: (**a**) HANb0, (**b**) HANb1, (**c**) HANb2, and (**d**) HANb3.

3D topographic AFM micrographs of the electrodeposits are presented in Fig. 5. The surface roughness of the HANb0 decreased with the incorporation of the Nb_2_O_5_ particles into the layers due to the generation of a compact microstructure, which contains smaller pores. HANb3 had the lowest surface roughness, ≈44% less than that of HANb0. Similar results have been reported by Fathyunes et al.^38^, where they showed that the surface roughness of the CaP electrodeposits fall from 450 to 234 nm with the included graphene oxide particles.

**Figure 6:**
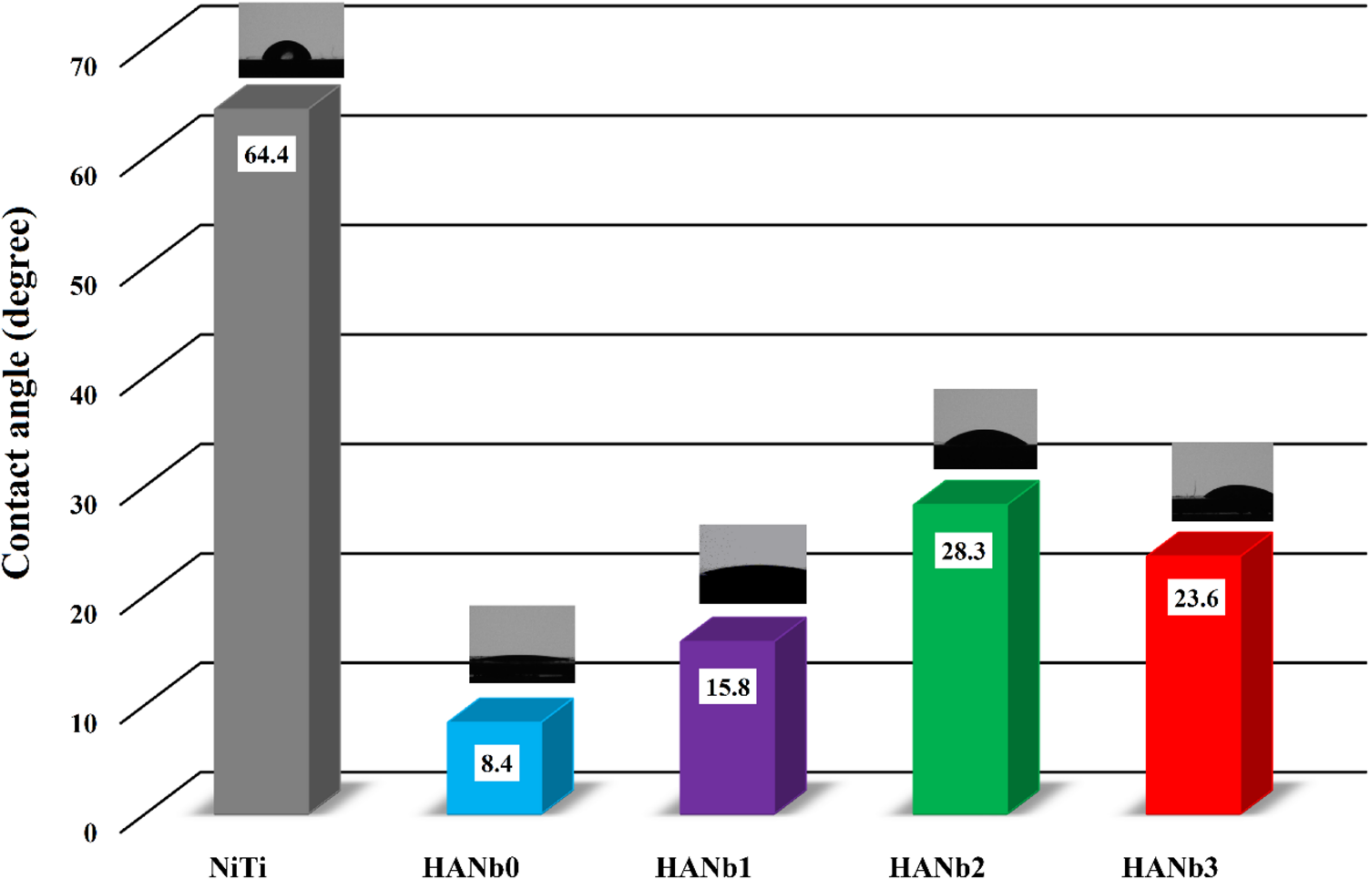
The contact angle values of the studied samples. The measurements were carried out using PBS drop.

The reason for an increase in surface roughness with the addition of excessive amounts of Nb_2_O_5_ particles can be attributed to the agglomeration of the rough ceramic particles, as mentioned in Fig. 5d). The co-deposited reinforcing particles also affect the crest-and-valley topography of the surface. While there are multiple crests and valleys in Fig. 5a), a more uniform topography with wider crests is observable in Fig. 5c). The influence of the included Nb_2_O_5_ particles on the surface wettability of the electrodeposited films is shown in Fig. 6.

Albeit all of the samples showed hydrophilic character, the application of the HAp-based layers on NiTi markedly encourages the surface wetting may be due to (i) the ionic character that of HAp ceramic film and (ii) the polar hydroxyl groups of the HAp. While the former provides a better substrate for the chemisorption of water-based liquid, the latter gives rise to coating/water molecules attraction by hydrogen bonding.

The contact angle value of the electrodeposited films increased with the inclusion of the Nb_2_O_5_ particles because the particles can partially inhibit the water molecules from penetrating the surface. It is to be noted that the superhydrophilic surfaces may have a harmful influence on cell adhesion and proliferation so that a moderately wettable surface can guarantee the best cell-material responses. In general, it can be concluded that the HANb2 layer suggests the best biological performance^39^.

Based on the Wenzel equation^40^, a higher surface roughness can rise the hydrophilicity of the hydrophilic surfaces (Ө < 90°). The results confirmed that the relationship between the surface roughness and wettability in this contribution complies with the Wenzel equation.

### In vitro immersion behavior

**Figure 7:**
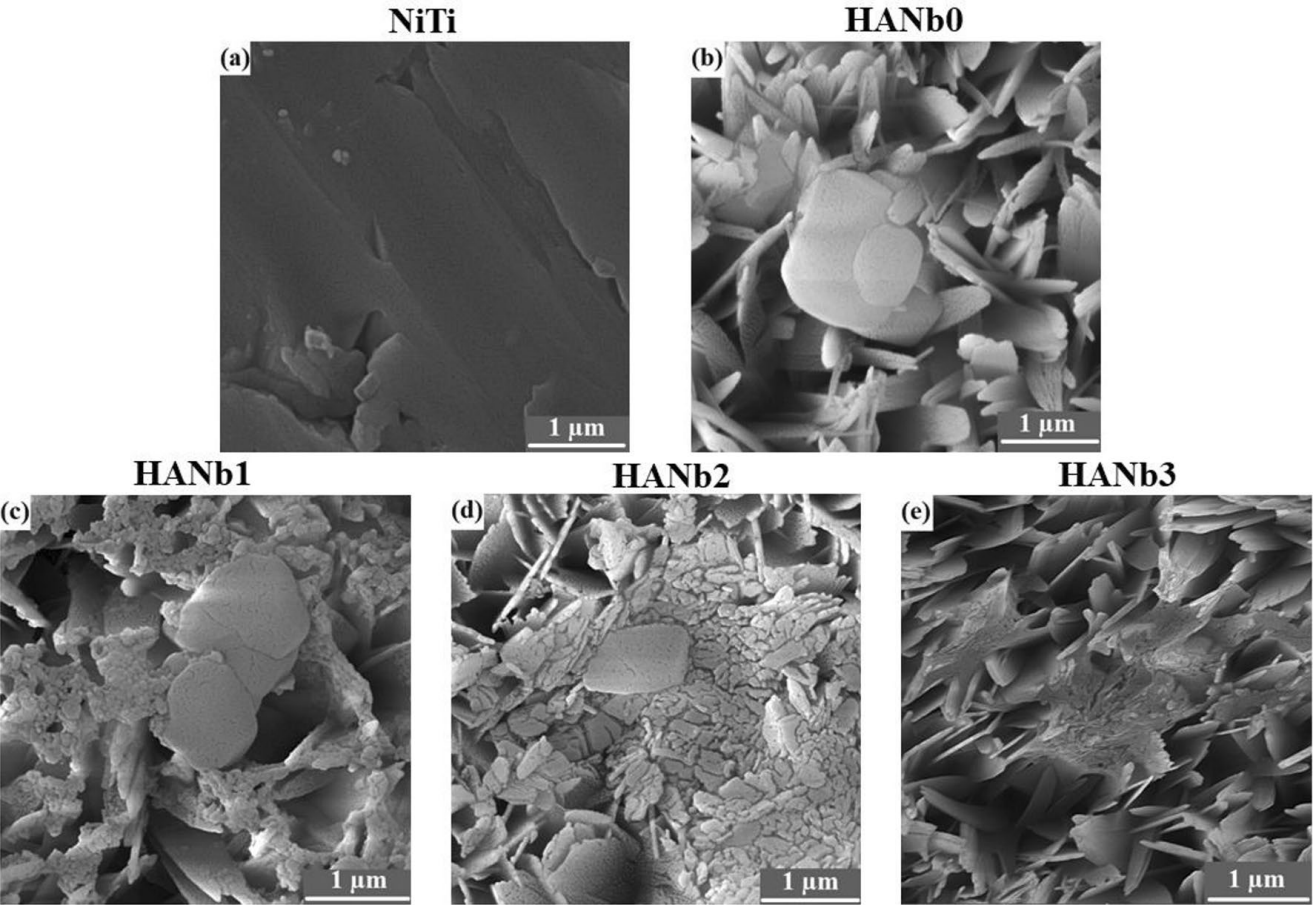
FESEM micrographs of the bare and coated samples after soaking in SBF for 7 days: (**a**) NiTi, (**b**) HANb0, (**c**) HANb1, (**d**) HANb2, and (**e**) HANb3.

The bone-forming ability of the biomaterials upon immersion in the SBF is one of the critical requirements for orthopedic applications since the formed apatite can act as an interface connecting the bone to the synthetic implant. The formation of the apatite upon soaking in SBF is a reliable marker for deciding whether the material is bioactive, even if the chemical composition of the apatite is not close to that of stoichiometric HAp. However, better bone-forming can be obtained when high amounts of stoichiometric HAp are formed on the biomaterial^14^. Figure 7 presents the FESEM micrographs of the bare and coated samples after soaking in SBF for 7 days.

Overall, the surface features, such as morphology, chemistry, topography, and particle size, are the important factors altering the in vitro bone-forming ability of the biomaterials^41^. Figure 7a) demonstrates that no apatite particles are formed on the surface of the bare NiTi, giving prominence to its poor bone-forming ability. The application of the HANb0 layer triggers, to some extent, apatite formation. The morphology of the formed apatite on HANb0 is polygonal (see Fig. 7b). The co-deposition of the Nb_2_O_5_ particles further stimulates the formation of tiny apatite particles; however, their morphology remains unchanged (see Fig. 7c,d). The tiny apatite particles formed on HANb1 and HANb2 layers provided a larger surface area, which guarantees better bonding with the surrounding tissue. The formation of smaller apatite particles correlates with a faster nucleation rate. It is well-established that the spherical particles that are formed after prolonged time immersion in SBF, i.e., more than 7 days, offer superior biomineralization^42, 43^. Figure 7e shows that there are fewer apatite particles that formed on the surface of the SBF-soaked HANb3 compared to the other composite electrodeposits. The enhanced bone-forming ability of HANb1 and especially HANb2 layers is related to their compact microstructure containing smaller crystallite size, the presence of appropriate amounts of Nb_2_O_5_ particles, and possessing minor amounts of DCPD phase. The reason why Nb_2_O_5_ particles stimulate apatite formation is ascribed to the low lattice mismatch between the particles and HAp^24^. Although the SBF solution contains corrosive elements, e.g., Cl^−^ ion, there is no sign of degradation on the surface of the films after 7 days of immersion, confirming the crystalline microstructure of the as-deposited coatings.

HAp dissolution and HAp precipitation are two main processes that take place when HAp-based coating is immersed in the SBF. It is well-established that a shift from dissolution to precipitation initiates after 5–7 days of immersion. During the first 5 days of immersion, the dissolution of HAp prevails over the HAp precipitation, leading to an increase in the local concentration of the Ca^2+^ ions in the vicinity of the coating, while the surface of the coating is negatively charged. If the concentration of the present Ca^2+^ and ${\mathrm{PO}}_{4}^{3-}$ ions in the bulk SBF also be taken into account, a relatively high concentration of the ions accumulates around the immersed layer. This can lead to the nucleation of a few nuclei due to the low supersaturation during the first 7 days. It can be inferred that the preferential growth of the crystals is responsible for the formation of polygonal apatite. With the prolonged immersion time, when a sufficient number of nuclei are formed, the growth stage will be the dominant process. Meanwhile, the morphology of the apatite particles is varied with the prolonged immersion time (more than 7 days) since there would be rapid nucleation due to the enhanced Ca^2+^ concentration and energy minimization principle^43, 44^. Results of SBF-soaking for 21 days published in our previous work^27^ prove the mentioned differences in the amount and morphology of the apatite particles formed after 7 and 21 of immersion in SBF. The Ca/P molar ratio and the amounts of Na and Mg elements in the microstructure of the coatings after immersion in SBF for 7 days are listed in Table 3.

**Table 3:** The Ca/P molar ratio and the amounts of Na and Mg elements in the microstructure of the coatings after immersion in SBF for 7 days.

Coating type	Na (wt.%)	Mg (wt.%)	Ca/P molar ratio
HANb0	0.36	–	1.55
HANb1	0.72	–	1.50
HANb2	1.43	0.15	1.52
HANb3	0.43	0.11	1.46

**Figure 8:**
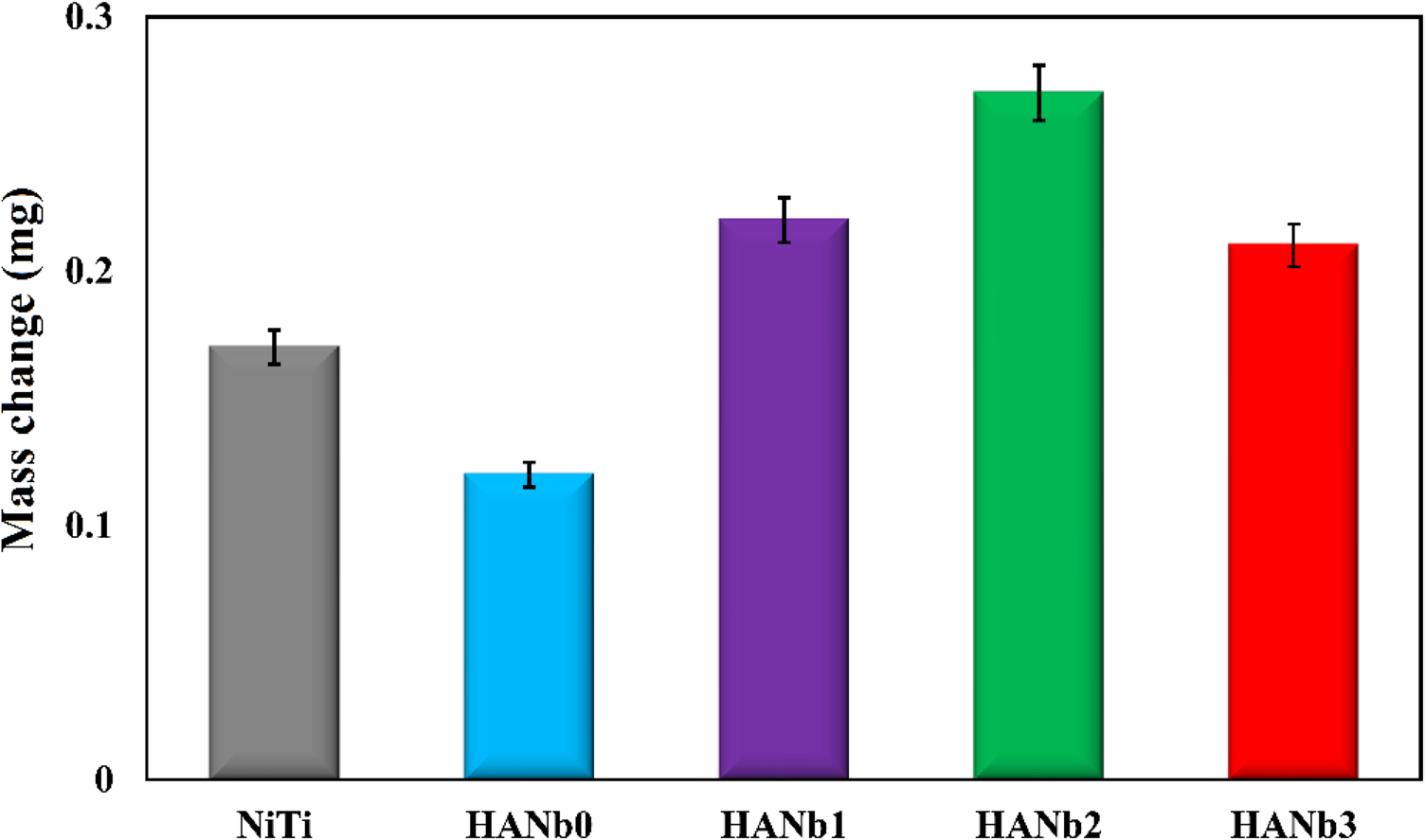
Mass change of the coatings after 7 days of immersion in SBF.

The Ca/P molar ratio of the SBF-soaked coatings is less than that of stoichiometric HAp, illustrating the formation of Ca-poor HAp. A possible reason is the incorporation of Na^+^ and Mg^2+^ ions into the HAp lattice, substituting for Ca^2+^ ions^45^. There is higher Na and Mg content in the structure of the composite coatings, in particular HANb2, which ensures their favorable biological performance since Na and Mg are essential elements for cell metabolism^46^. A comparison between the amount of these elements after 7 and 21 days of immersion in SBF shows that their content in the coatings increases directly with the immersion time. The mass change of the coatings after 7 days of immersion in SBF is presented in Fig. 8.

It is seen that a slight increase in the mass is obtained due to the formation of the apatite particles. However, the degree of the mass increment is lower than that obtained after prolonged immersion time, i.e., 21 days. The precipitation of the existing salts in SBF on the bare NiTi is responsible for its increased mass.

Although both SBF and PBS acellular media are widely employed to study the in vitro immersion behavior and, to some degree, simulate the body fluid condition and corrosiveness of the fluid, there are significant differences between their chemical compositions (see Supplementary Table I for the chemical compositions of SBF and PBS acellular media). The lack of Ca^2+^ ions in the composition of PBS solution is one of the highlighted differences between the two media, which restricts apatite formation on the biomaterials upon immersion in PBS, even for prolonged times^47^.

**Figure 9:**
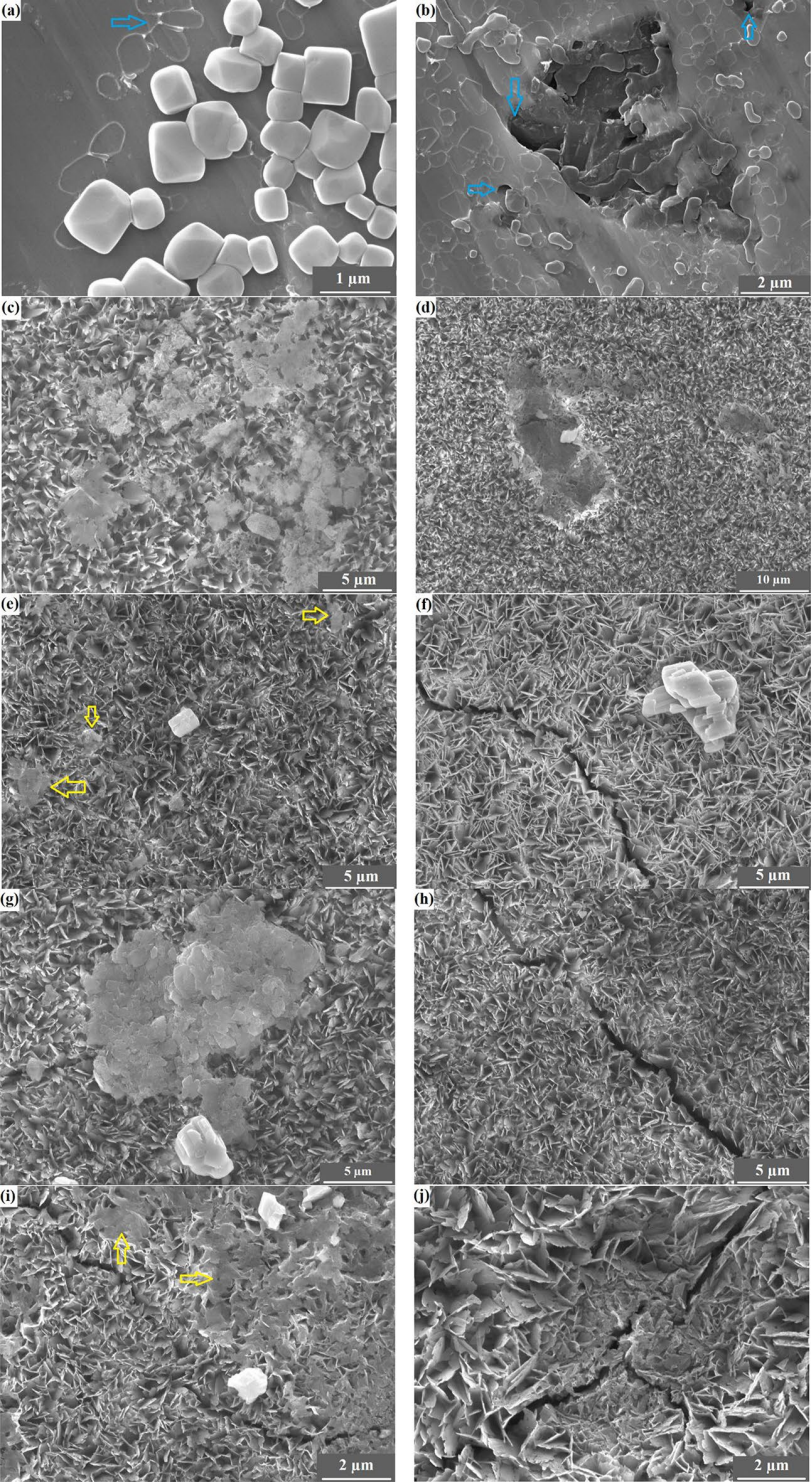
The FESEM images of the specimens soaked in PBS for 30 days: (**a**,**b**) NiTi, (**c**,**d**) HANb0, (**e**,**f**) HANb1, (**g**,**h**) HANb2, and (**i**,**j**) HANb3.

The FESEM images of the 30 days PBS-soaked specimens are indicated in Fig. 9. Figure 9a shows that there are NaCl salts accumulated throughout the surface of bare NiTi (EDS elemental mapping and EDS line scan profile elemental distribution is provided in Supplementary Fig. III). When the PBS medium evaporates, Cl^-^ ions can be oversaturated and a higher concentration of the ions accumulated on the bare NiTi. The precipitated Cl^-^ ions can attack the formed oxide layer on the NiTi, causing pitting corrosion, as indicated by blue arrows in Fig. 9b). Such a corrosion attack can lead to serious health concerns since toxic Ni^2+^ ions are released as a result of NiTi degradation.

A quite different surface morphology from that obtained for bare NiTi is observable for the HAp-based coated NiTi, where Ca-poor apatite particles are formed on the coatings. Multiple apatite grains form on the PBS-soaked HANb0 (see Fig. 9c); however, there are clear signs of corrosion attacks, such as microcracks and coating delamination, on the surface (see Fig. 9d). It seems that the porous HAp top layer is damaged, and the underlying dense HAp still remains intact. Albeit there are apatite particles formed on the surface of HANb1, HANb2, and HANb3 samples, only a few defects, e.g., microcracks, are seen, demonstrating the enhanced corrosion protection with the included Nb_2_O_5_ reinforcing phase (Fig. 9cj). Overall, the HANb2 sample stimulates the most favorable in vitro immersion behavior since there is the highest number of apatite particles formed on its surface. The co-deposited Nb_2_O_5_ particles, in an optimum level, enhance the apatite formation since the particles can serve as potential nucleation sites^48^. The addition of an excessive reinforcing phase led to poor corrosion resistance as the coating is strongly degraded after 30 days of immersion in PBS (Fig. 9j). The coatings preserve their integrity after 30 days of immersion in the corrosive PBS solution without detachment or complete degradation, indicating their excellent stability. While Duarte et al.^49^ have indicated that the electrochemically deposited HAp layer on Ti-13Nb-13Zr was completely degraded after PBS soaking for 49 days, the published results by Iskandar et al.^50^ showed the detachment of a part of the HAp film after 28 days of immersion.

**Figure 10:**
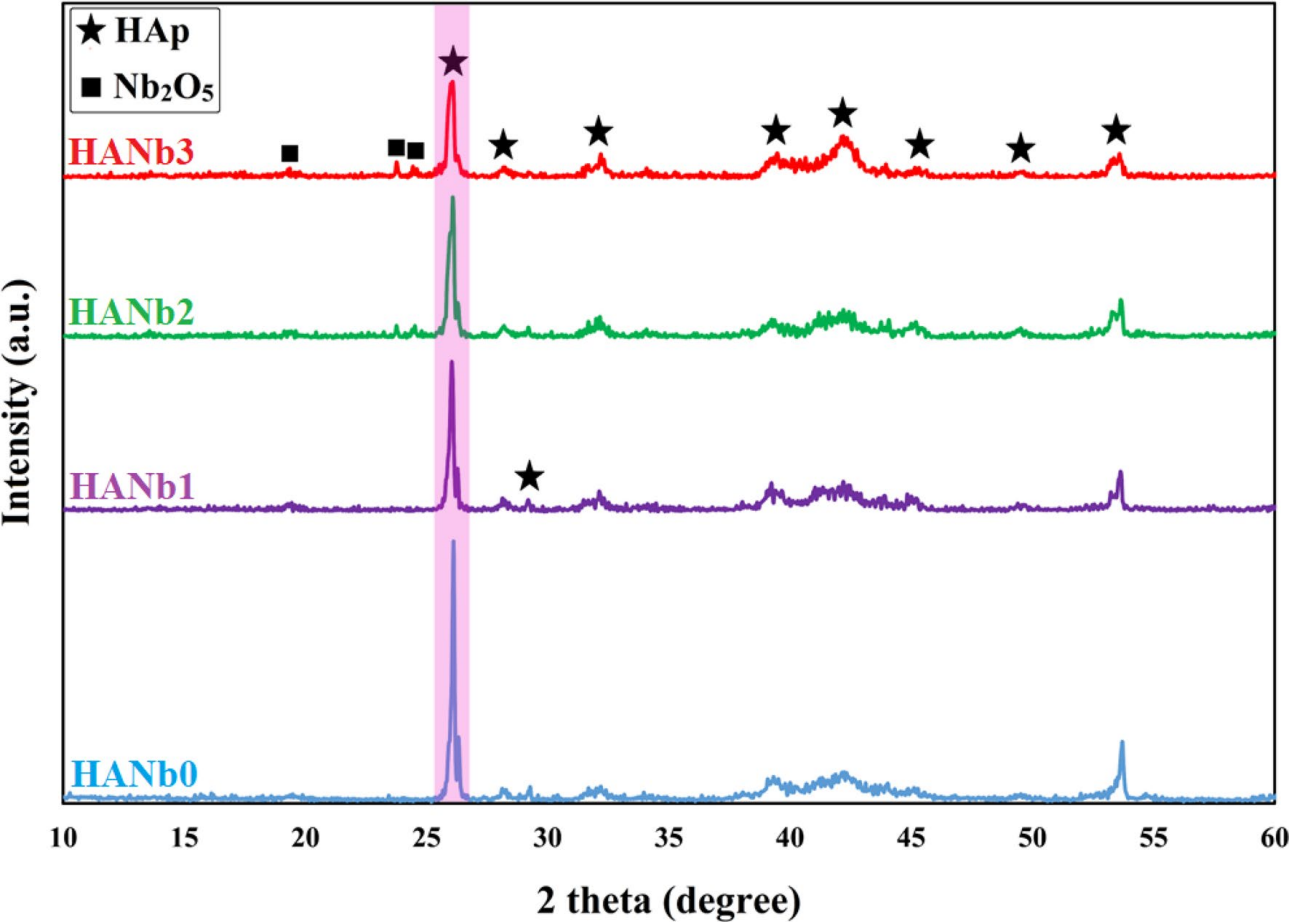
XRD patterns of the electrodeposited layers upon immersion in PBS for 30 days.

The mechanism governing the apatite mineralization on the coated samples upon immersion in solution without Ca^2+^ ions is the dissolution of the immersed coatings, where the Ca^2+^ ions released in PBS and the surface of the coating becomes negatively charged; therefore, the ionic exchange takes place in the PBS medium. Simply put, fierce competition between the dissolution and precipitation of apatite is in progress during the PBS immersion period so that the coatings gain a big part of the lost mass by the formation of the new apatite grains. In general, the reported results on the possibility of apatite formation on the HAp coated-implants upon immersion in PBS are different. For instance, Vranceanu et al.^47^ have illustrated that there is no apatite formed on Ag-doped HAp layers after 21 days of immersion in PBS, while a progressive increase in the amount of formed apatite on CaP-chitosan coating within the 15 days immersion period has been reported by Wu et al.^51^. Taking the findings of the present assay into consideration, it can be inferred that the physicochemical properties of the coatings and the type of media profoundly influence the in vitro immersion behavior. The XRD patterns of the electrodeposited layers upon immersion in PBS for 30 days are illustrated in Fig. 10. (The XRD pattern of the PBS-soaked bare NiTi is presented in Supplementary Fig. IV). The XRD patterns of the immersed coatings comprise typical crystalline HAp peak, and there is no change in the main growth orientation of the crystals, i.e., the c-axis.

Comparing the patterns with those obtained for the as-deposited coatings in our previously published paper^27^, it can be concluded that there is no change in the position of the peaks and no new peak has appeared, while the intensity of HAp peak at 2Ө ≈ 26° is markedly decreased. The findings suggest that the apatite dissolution prevails over the apatite mineralization within the 30 days immersion period. However, it should be noted that the released components are Ca^2+^ and phosphate ions, which can be used by osteoblasts for forming bone tissue. Table 4 outlines the Na weight percent and Ca/P molar ratio of the PBS-soaked layers obtained by EDS analysis.

**Table 4:** Na weight precent and Ca/P molar ratio of the PBS-soaked layers (for 30 days) obtained by EDS analysis.

Coating type	Na (wt%)	Ca/P molar ratio
HANb0	0.67	1.36
HANb1	0.65	1.39
HANb2	1.13	1.41
HANb3	1.07	1.38

**Figure 11:**
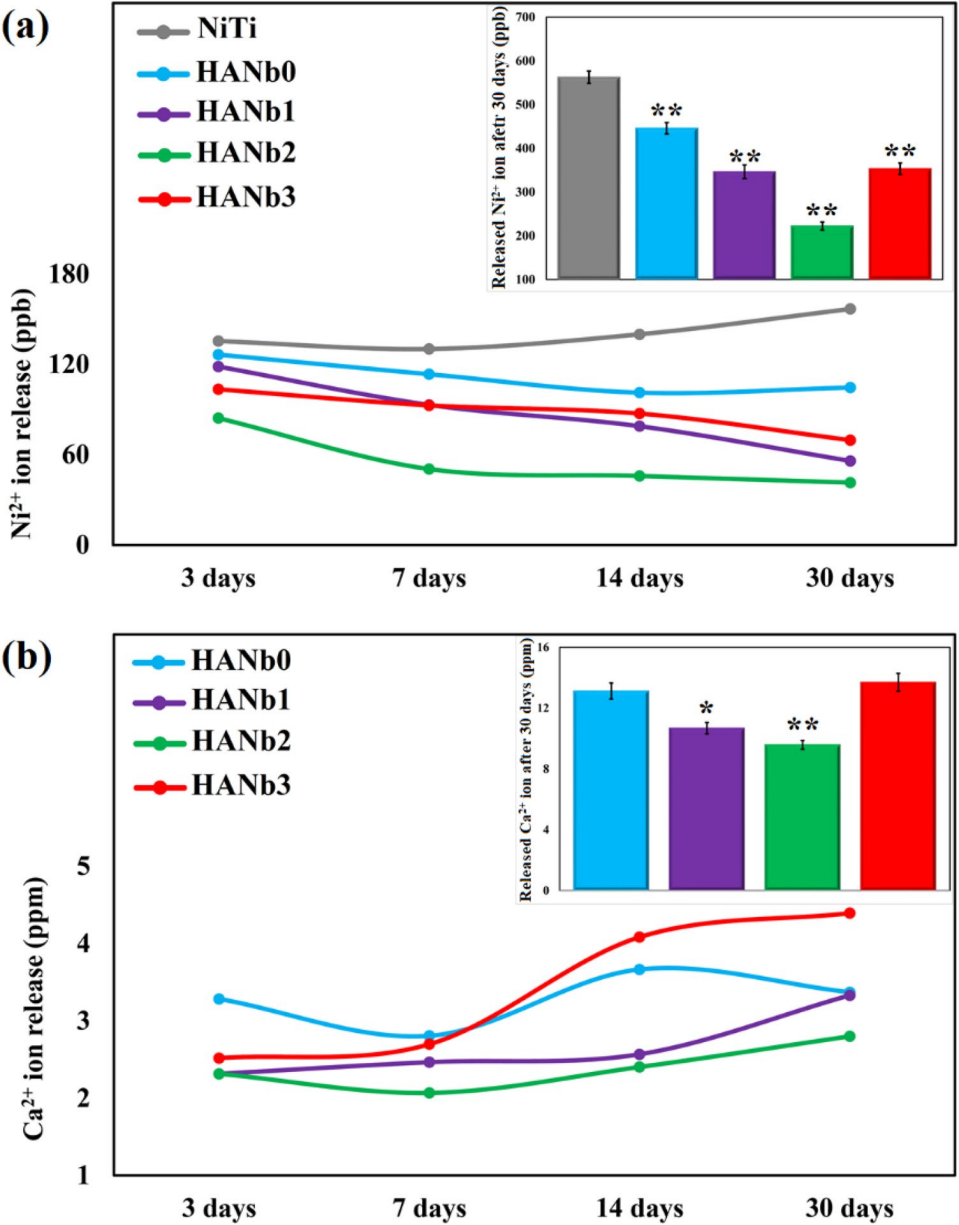
Concentration of the: (**a**) released Ni^2+^ (**p < 0.0001 with respect to bare NiTi) and (**b**) Ca^2+^ ions (*p = 0.0001 and **p < 0.0001 with respect to HANb0) upon 30 of immersion in PBS at 37 °C. The windowed images at top right corner show the total concentration of the corresponding ion after 30 days.

The uptake of Na is beneficial for the in vivo applications of the films since Na is a necessary mineral for the metabolism of living cells and for stimulating eukaryotic cell growth. The results show the formation of Ca-deficient apatite. There is a slight difference between the Ca/P molar ratio of the layers. Figure 11 illustrates the concentration of the released Ni^2+^ and Ca^2+^ ions upon 30 of immersion in PBS at 37 °C. The surface modification of the NiTi with Nb_2_O_5_-containing HAp coatings led to a statistically significant decrease in the Ni^2+^ ion leaching. During the first 3 days of PBS soaking, the concentration of released Ni^2+^ ions from the surface of bare NiTi is almost close to the coated samples; however, an opposite long-term trend in the ion leaching between the bare and coated samples is observable, where the amount of the released Ni^2+^ ions from the surface of bare NiTi is about three times greater than that of Nb_2_O_5_-containing HAp coatings within the time interval of 14–30 days.

The surface of NiTi is naturally oxidized to form titanium-containing oxides. While a Ti–rich oxide top layer is formed on the NiTi by out-diffusion of Ti, the chemistry of the underlying surface is Ni-rich. The formed oxide layer can impede the Ni^2+^ leaching during the initial few days; however, since this thin film is unstable and defective, the long-term in vivo use of bare NiTi faces serious challenges^52^. Simply put, the Ni^2+^ ions can leach through the titanium-containing oxide layer when it is damaged by the corrosive ions in the physiological medium, e.g., Cl^-^ and dissolved oxygen. The corrosion products formed over the bare NiTi after potentiodynamic polarization assay in the potential range of − 300 to 1200 mV *vs.* the OCP in the Ringer’s solution at 37 °C were Ti-containing oxides^53^. The results of the present survey confirm the hypothesis, where the concentration of the released Ni^2+^ ions increases with the prolonged immersion time. The FESEM image of the PBS-immersed NiTi clearly shows the pitting corrosion on the surface of the NiTi (see Fig. 9b). On the other hand, the electroplated coatings provide efficient surface protection so that a remarkable decrease in the amount of the released Ni^2+^ ions is obtained. The inclusion of the Nb_2_O_5_ particles improved the corrosion resistance of the HAp coating. HANb2 coating showed the least Ni^2+^ ions release, which highlights the importance of the addition of proper amounts of the reinforcing particles rather than high or excessive amounts. The Nb_2_O_5_-reinforced films benefit from a more compact microstructure, higher density, smoother surface, the ability of biomimetic apatite formation upon immersion in PBS, and the presence of dispersed Nb_2_O_5_ particles that can act as barriers against corrosive attacks. In addition, there is much less amount of the accumulated Cl^-^ ions on the surface of the coated samples. The reactions that may be occurred as a result of Cl^-^ ion adsorption on the surface are outlined below^54^:

1 $${\text{Ni }} + {\text{ 2Cl}}^{ - } = {\text{ NiCl}}_{{2}} + {\text{ 2e}}^{ - }$$

2 $${\text{Ni }} + {\text{ H}}_{{2}} {\text{O }} = {\text{ NiO }} + {\text{ 2H}}^{ + } + {\text{ 2e}}^{ - }$$

3 $${\text{NiO }} + {\text{ 2Cl}}^{ - } + {\text{ 2H}}^{ + } = {\text{ NiCl}}_{{2}} + {\text{ H}}_{{2}} {\text{O}}$$

Zhang et al.^54^ have reported that the deposition of sol–gel derived HAp coating on NiTi can seriously diminish the concentration of the released Ni^2+^ ions. A higher Ni^2+^ ion leaching has been detected when the coating comprises higher porosity content.

Overall, a feasible and cost-effective strategy for the clinical use of the HAp-Nb_2_O_5_ coated-NiTi hybrid system is the immersion of the samples in SBF or PBS for 7 days before implantation in vivo since a noticeable decrease in the Ni ion leaching is achieved.

Figure 11b indicates that the concentration of the released Ca^2+^ ion increased with the immersion time, indicating that the dissolution prevails over the biomineralization. The highest Ca^2+^ ion concentration is registered for the HANb3 sample, which can be interpreted by the synergistic role of Ca ion release from the coating due to the occurred corrosion and the dissolution of newly-formed apatite. On the other hand, the HANb2 layer, which contains an optimum content of Nb_2_O_5_ reinforcing phase, offers the lowest dissolution in PBS during the immersion period. The favorable biomineralization originating from the presence of Nb_2_O_5_ particles ensures the formation of a significant amount of apatite particles^24^.

When immersed in a corrosive PBS medium, the application of HAp-Nb_2_O_5_ layers not only suppresses the Ni^2+^ release but also contributes to the formation of biomimetic apatite particles since the beneficial Ca^2+^ ions release upon corrosion.

### Antibacterial activity

**Figure 12:**
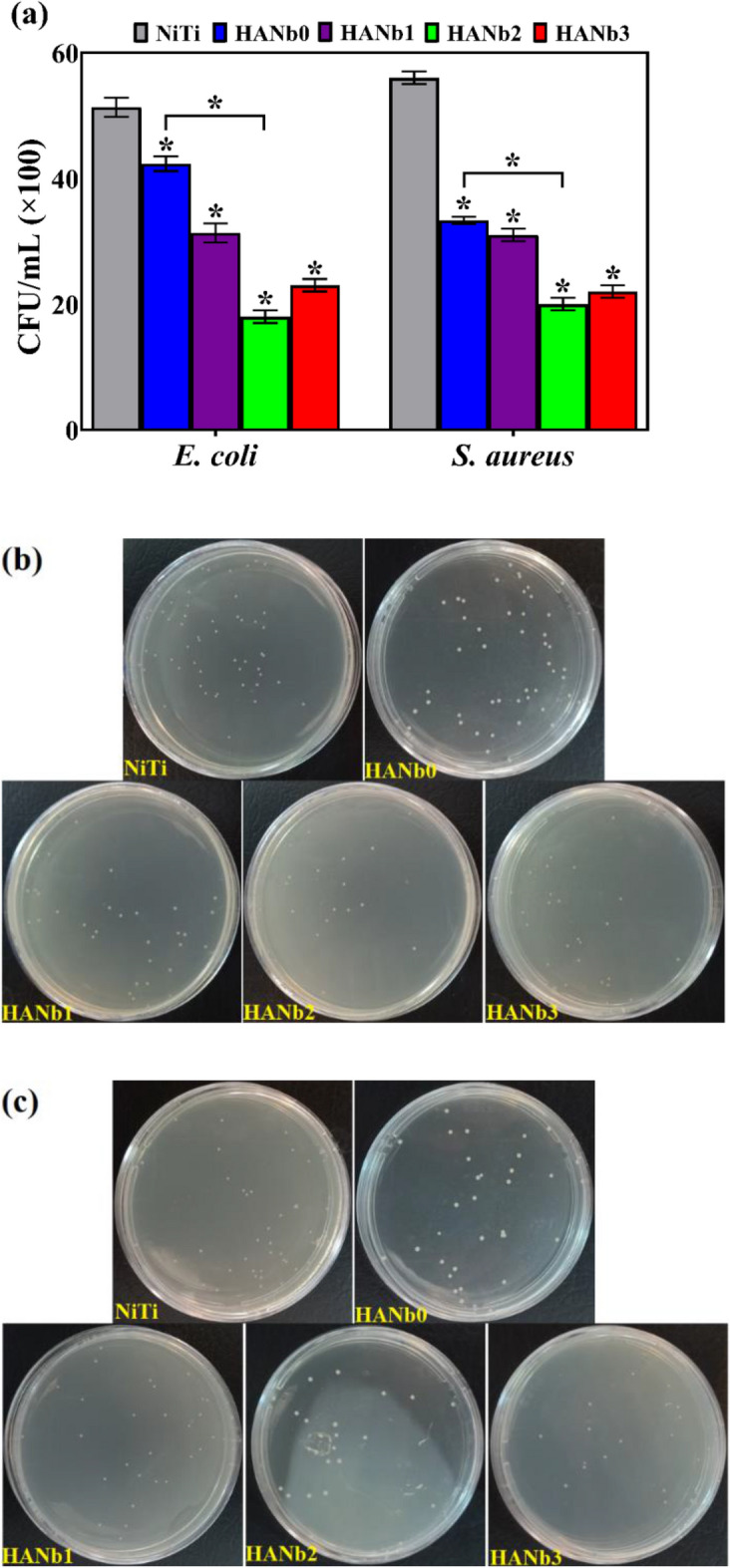
The quantitative results of plate-counting assay against both *E. coli* and *S. aureus* bacteria (*p < 0.0001 with respect to bare NiTi), and optical images of the formed (**b**) *E. coli* and (**c**) *S. aureus* bacterial colonies on agar plates.

Bacterial adhesion is the first and may be the most important factor, causing post-implantation and device-associated infections, which can become chronic. A factor that can contribute to the infection diffusion is the formation of bacterial biofilms, communities of microorganisms that are more resistant to treatment in comparison to the planktonic bacteria. Bacterial adhesion can be affected by a variety of parameters, including bacterial properties, surface features, and environmental variables^55^. To sum up, post-implantation bacterial infections put an additional health and economic burden on the patient; therefore, it is necessary to change the surface properties of the synthetic implant to address this hurdle. The antibacterial activity of the studied samples was evaluated against the Gram-negative *E. coli* and the Gram-positive *S. aureus* bacteria. The mentioned bacteria are serious causes of hospital-acquired infections, so they take up dominant positions in clinical infections that need to be treated^56^. The antibacterial activity of the samples was evaluated by plate-counting and MTT metabolic assays. The former was performed to evaluate the antibacterial properties against planktonic bacteria that were incubated on the samples and to investigate whether the release of ions was effective, while the latter was carried out to assess the anti-adhesive activity of the surfaces. The results of the plate-counting assay and the optical images of the agar plates containing bacterial colonies are illustrated in Fig. 12. The results demonstrate that the HANb2 offers the highest antibacterial activity against both *E. coli* and *S. aureus* bacteria.

The Ni^2+^ ion release from bare NiTi is responsible for its antibacterial activity due to the genome damage induced by the ions^57^. It is well-accepted that the Ni^2+^ ion suggests antibacterial performance even though it causes health concerns, such as allergies^11, 15, 58^. The results showed that the deposition of the HANb0 layer has a positive contribution to the antibacterial activity of the NiTi implant against both *E. coli* and *S. aureus*. The mechanism governing the HANb0 antibacterial performance is the direct contact between the HAp nanocrystals and microorganisms^59^. The HANb2 coating provided the highest activity against both *E. coli* and *S. aureus* due to its superior surface chemistry and topography, namely presence of Nb_2_O_5_ particles, lower surface roughness, and higher hydrophilicity. The included Nb_2_O_5_ particles provide additional surface area that can be a potential factor in killing bacteria. As shown in TEM micrographs of HANb2 film (see Fig. 2b), there are numerous nano-sized Nb_2_O_5_ crystallites that either dispersed all over the coating or located on the agglomerated particles (see Supplementary Fig. V for the TEM images of as-purchased Nb_2_O_5_ particles)^60, 61^.

Although the information on the antibacterial activity of Nb_2_O_5_ is scarce^62^, the obtained results proved that the addition of Nb_2_O_5_ particles can improve the antibacterial activity of the HAp. The quantitative results of the antibacterial rate are listed in Supplementary Table II. Under GB/T 20944.3 standard, a biomaterial is considered an antibacterial material if it possesses an antibacterial rate of 70% against both *E. coli* and *S. aureus* pathogenic bacteria^59^. Although the obtained antibacterial rates for the majority of the coated samples in this work did not exceed 70%, the activity of HANb2 coating is higher than the ideal value, showing the marked antibacterial efficiency of the Nb_2_O_5_ phase and highlighting that the prime importance of R & D works to further rise the antibacterial activity of Nb_2_O_5_-reinforced HAp films. There are several ongoing projects by our research group in which Ag and ZnO dopants are added to the HAp-Nb_2_O_5_ electrodeposits to further rise their antibacterial behavior.

**Figure 13:**
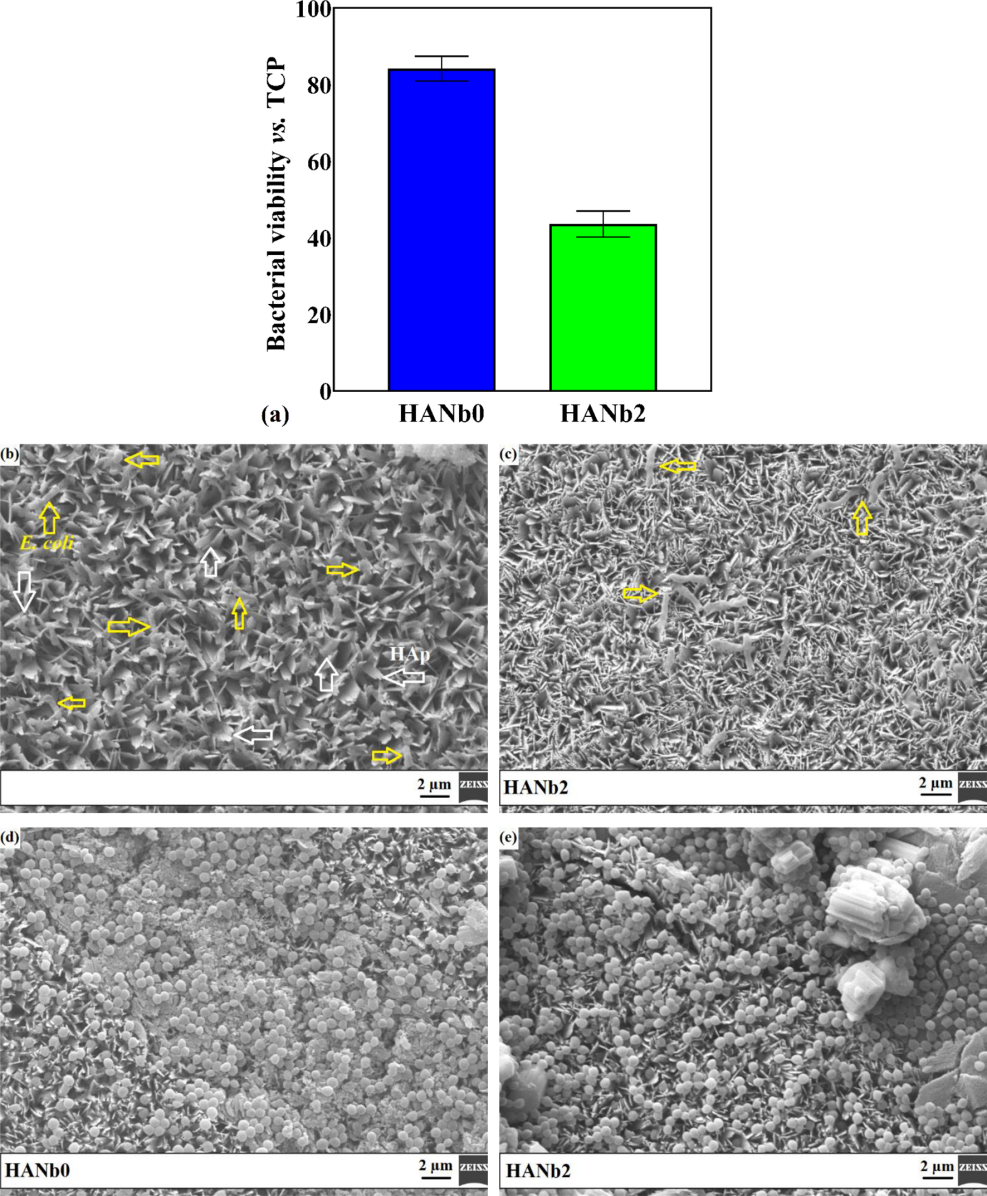
(**a**) MTT results of the antibacterial activity of HANb0 and HANb2 layers against planktonic adherent *E. coli* and *S. aureus*; *E. coli* bacteria morphology on (**b**) HANb0 and (**c**) HANb2; and *S. aureus* bacteria morphology on (**d**) HANb0 and (**e**) HANb2.

The anti-adhesive action of HANb0 and HANb2 was assessed through MTT colorimetric assay to illustrate the effect of the electrodeposited HAp coating and included Nb_2_O_5_ particles on the anti-adhesive performance of the NiTi implant (see Fig. 13). It is to be noted that bare NiTi was served control in this experiment.

Figure 13a reveals a substantial fall in the viability of the planktonic adherent *E. coli* bacteria with the co-deposition of the Nb_2_O_5_ reinforcing phase due to the decreased surface roughness and increased hydrophobicity (see Fig. 6)^63^. Figure 13b,c) clearly illustrate the rod-shaped morphology of the existing *E. coli* bacteria on the surface (yellow arrows). Additionally, the HAp crystals are shown by white arrows. Since there is a higher porosity content in the surface of the HANb0 sample, some of the adherent bacteria may stay inside the pores. A high-magnification view of the HANb0 image with larger size is illustrated in Supplementary Fig. VI to provide a clearer view of the *E. coli* bacteria over the surface. In conclusion, the Nb_2_O_5_ particles not only restrict the *E. coli* bacteria adhesion but also kill, to some extent, the bacteria.

Compared to bare NiTi, there are slightly lower, by ≈20%, adherent *E. coli* and *S. aureus* bacteria on the surface of HANb0. The obtained results are in agreement with the literature^64^. Indeed, the hydroxyapatite crystals are effective in reducing bacterial viability since they cause damage to the cell membrane due to their abrasive morphology.

It is found out that the HAp-based composite film has more influence on Gram-negative bacteria than Gram-positive ones, which may be due to the bacteria’s cell wall properties, such as wall components, charge, and thickness. The bacterial cell wall of *E. coli* (7–8 nm) is much thinner than that of *S. aureus* (20–80 nm)^60, 65^. The obtained results in this work comply with those reported in the literature^60, 66^. Sivaraj et al.^60^ have illustrated that the antibacterial activity of HAp coating produced by spray pyrolysis technology improved with the addition of MWCNT. The ZOI of the MWCNT-reinforced HAp films against Gram-negative is larger than that obtained for Gram-positive.

The mechanism of how the included Nb_2_O_5_ particles affect antibacterial activity may be attributed to a change in electrostatic interactions between the coating and cell wall of the bacteria and the generation of reactive oxygen species (ROS) as a result of the interaction between the particles and topoisomerase^67–69^. Afifi et al.^70^ have observed a noticeable enhancement in the antibacterial activity of the HAp powder with the addition of Nb_2_O_5_ particles due to the contribution of the dopant to the ROS generation and the bacterial DNA damage caused by the particles.

Overall, the electroplated composite coatings in this work serve as passive coatings that kill the bacteria upon contact. The enhanced antibacterial performance of the active coatings lies in releasing antibacterial agents^71^. However, the beneficial contribution of the released Nb^5+^ ions from the composite coatings, which is < 50 and < 30 ppb during the 24 h of immersion in SBF and PBS, respectively, should not be neglected. Nb^5+^ ions can promote the performance of immunity cells, e.g., macrophages and neutrophils; therefore, they serve as a booster of immunization. The possible reason for addressing the decreased antibacterial performance with the higher content of the included Nb_2_O_5_ particles, i.e., HANb3, is attributed to the conversion of the NbO_6_ structural units into NbO_4_ ones^72^.

## Conclusions

In this work, HAp-Nb_2_O_5_ composite layers with different levels of Nb_2_O_5_ particles were electrodeposited on NiTi. The stress has been put on evaluating the role of Nb_2_O_5_ particles in realizing the surface characteristics, in vitro immersion behavior, Ni^2+^ ion leaching, and antibacterial activity of the pure HAp coating. The key conclusions are listed as follows:

i. A more compact layer with lower surface roughness is obtained with the co-deposition of the Nb_2_O_5_ particles.
ii. The applied HANb0 coating drastically decreased the contact angle of the NiTi biomaterial. The Nb_2_O_5_-reinforced layers showed higher contact angle values, still remaining hydrophilic, which is definitely beneficial for the biological and antibacterial characteristics of the layers.
iii. Unlike bare NiTi, the coated samples exhibited encouraging in vitro biomineralization by stimulating the formation of Ca-poor apatite upon immersion in the SBF. The HANb2 coating benefited from the advantage of uptaking Na and Mg elements upon the short-period SBF soaking.
iv. The composite layers, especially HANb2, showed high stability during the 30 days of immersion in corrosive PBS. Meanwhile, the HAp-based coatings triggered the apatite formation in contrast with the bare NiTi, which was degraded with pitting corrosion.
v. The Ni ion leaching from the NiTi surface is highly suppressed with the applied surface modification technique. The application of the HANb2 coating guarantees the least Ni ion leaching, which is by far less than the limit threatening human health.
vi. The antibacterial activity of pure HAp against Gram-negative and Gram-positive pathogenic bacteria was increased with the included Nb_2_O_5_ particles. The HANb2 layer showed the highest antibacterial performance against both *E. coli* and *S. aureus* bacteria.
vii. The Nb_2_O_5_-reinforced composite coatings bear distinct advantages over the bare and HAp-coated NiTi, opening up new opportunities to develop surface finishing of NiTi for orthopedics. The addition of a third phase, having prominent antibacterial activity, is believed to further improve the overall efficiency of the layers beyond that already available.

### Supplementary Information

Supplementary Information.

## Data Availability

The datasets used and/or analyzed during the current study are available from the corresponding author on reasonable request.

## Abbreviations

AFM: Atomic force microscopy
CFU: Colony-forming unit
DCPD: Dicalcium phosphate dihydrate
EDS: Energy dispersive X-ray spectroscopy
FAT: Fixed analyser transmission
FESEM: Field emission scanning electron microscopy
HAp: Hydroxyapatite
ICP-MS: Inductively coupled plasma mass spectrometry
ICP-OES: Inductively coupled plasma optical emission spectroscopy
MTT: 3-(4,5-Dimethylthiazol-2-yl)-2,5-diphenyl tetrazolium bromide
MWCNT: Multi-walled carbon nanotube
PBS: Phosphate-buffered saline
PC: Pulse current
SBF: Simulated body fluid
UV: Ultraviolet
XPS: X-ray photoelectron spectroscopy
XRD: X-ray diffraction
ZOI: Zone of inhibition
