# Supplementary material for: Enhanced in vitro immersion behavior and antibacterial activity of NiTi orthopedic biomaterial by HAp-Nb2O5 composite deposits
Source: Sci Rep. 2023 Sep 25;13:16045. doi: 10.1038/s41598-023-43393-3 (PMC10520115; doi:10.1038/s41598-023-43393-3)
Supplement: Supplementary file 1 — Supplementary Information. [file 41598_2023_43393_MOESM1_ESM.docx]

**Enhanced *in vitro* immersion behavior and antibacterial activity of NiTi orthopedic biomaterial by HAp-Nb_2_O_5_ composite deposits**

Mir Saman Safavi^1,2*^, Jafar Khalil-Allafi^1*^, Elisa Restivo^2,3^, Arash Ghalandarzadeh^4^, Milad Hosseini^1^, Giacomo Dacarro^5^, Lorenzo Malavasi^5^, Antonella Milella^6^, Andrea Listorti^6^, Livia Visai^2,3*^

^1^Research Center for Advanced Materials, Faculty of Materials Engineering, Sahand University of Technology, Tabriz, Iran, P.O. Box: 51335-1996.

^2^Molecular Medicine Department (DMM), Center for Health Technologies (CHT), UdR INSTM, University of Pavia, Viale Taramelli 3/B, 27100 Pavia, Italy.

^3^Medicina Clinica-Specialistica, UOR5 Laboratorio di Nanotecnologie, ICS Maugeri, IRCCS, 27100 Pavia, Italy.

^4^School of Metallurgy and Materials Engineering, Iran University of Science and Technology, Tehran, Iran.

^5^Department of Chemistry and INSTM, University of Pavia, Viale Taramelli 16, 27100 Pavia, Italy.

^6^Department of Chemistry, University of Bari Aldo Moro, Via Orabona 4, 70125 Bari, Italy.

^*^Corresponding authors: Prof. Jafar Khalil-Allafi (jallafi@yahoo.de), Prof. Livia Visai (livia.visai@unipv.it), and Mir Saman Safavi (samansafavi1992@gmail.com)

**Supplementary figures**


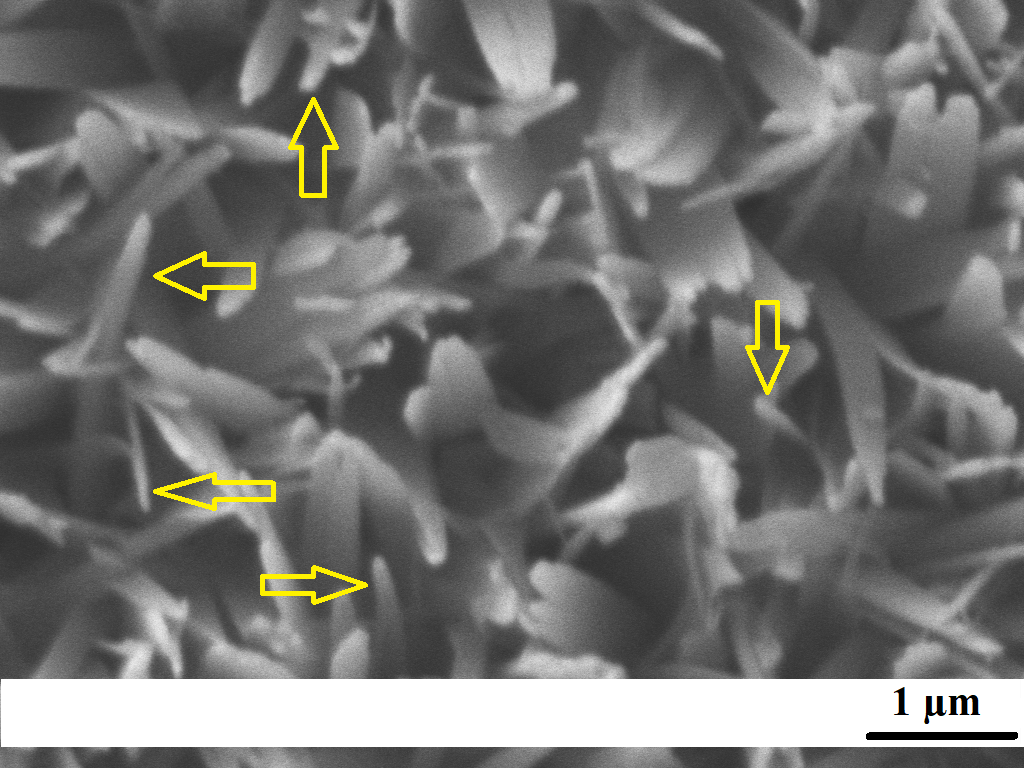


**Figure I.** High-magnification SEM image of HANb0 film. The arrows show the presence of needle-like crystals.


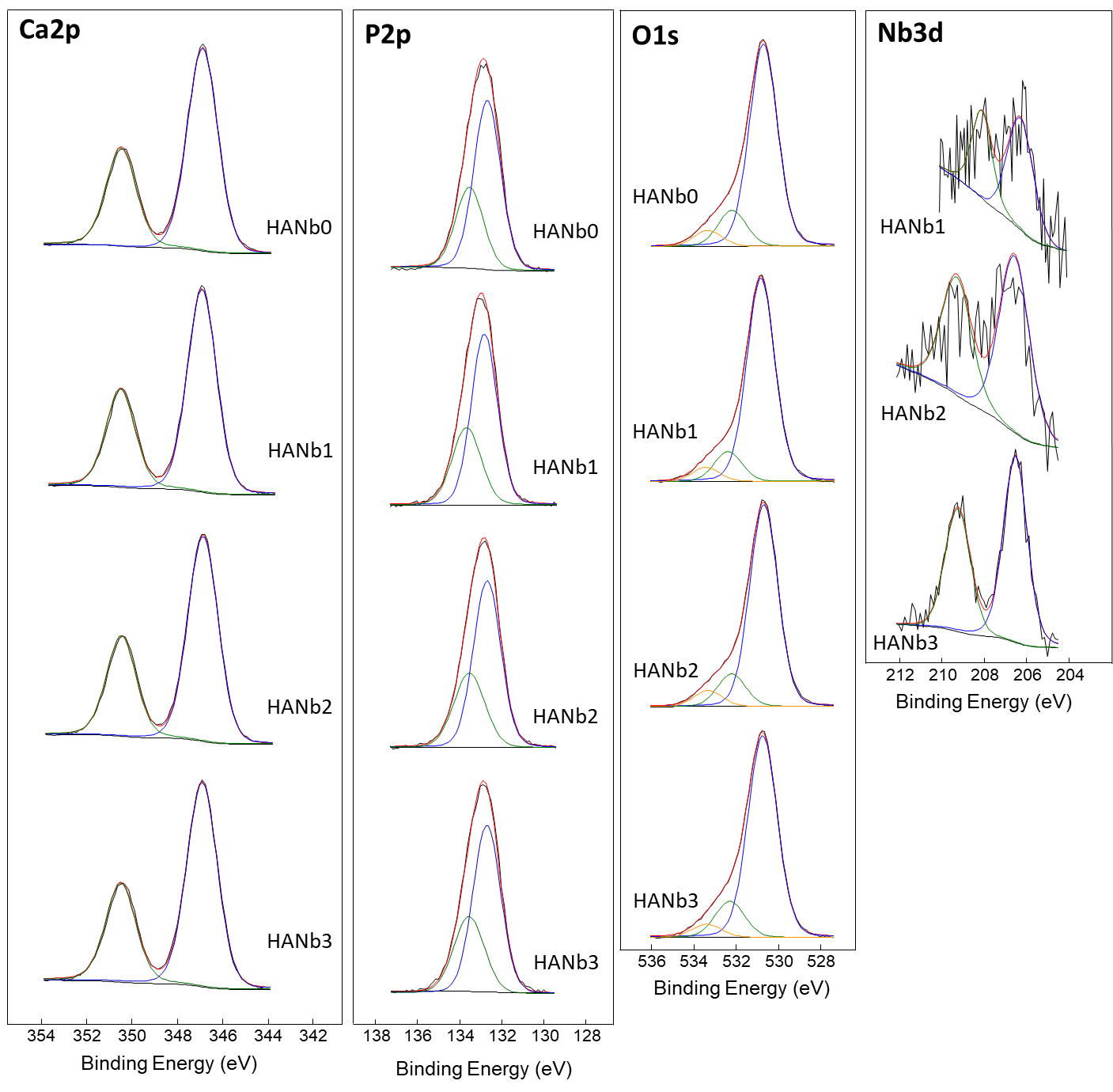


**Figure II.** Comparison of the XPS high-resolution spectra for HANb0-HANb3 coatings. Peak assignments are analogous to those of Fig. 4 in the main text.


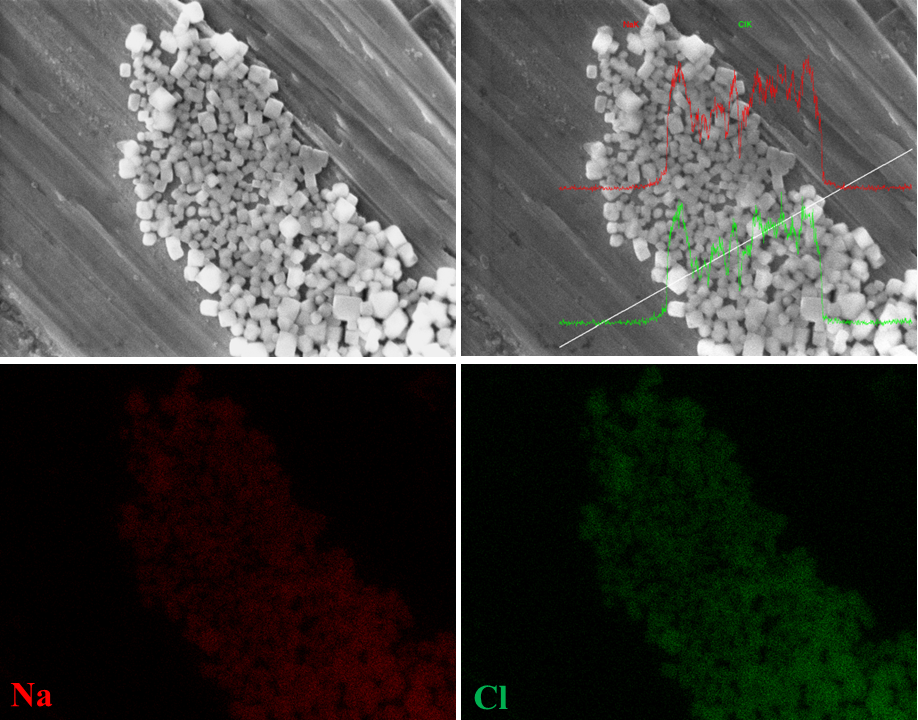


**Figure III.** EDS line scan elemental distribution profile and EDS elemental mapping of the bare NiTi after 30 days of soaking in PBS.


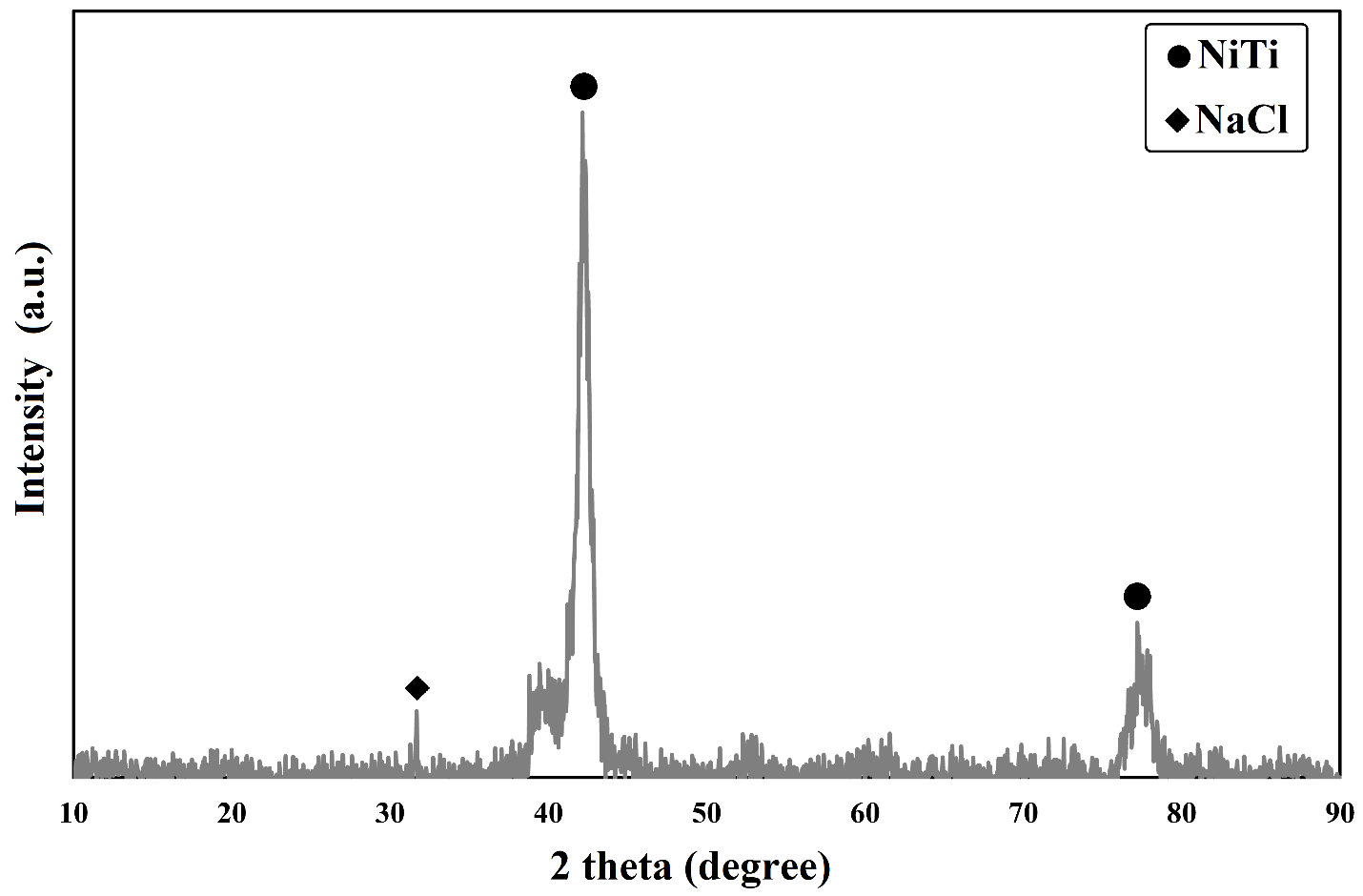


**Figure IV.** XRD spectrum of the bare NiTi after immersion in PBS for 30 days.





**Figure V.** Bright-field TEM image of the as-purchased Nb_2_O_5_ particles.


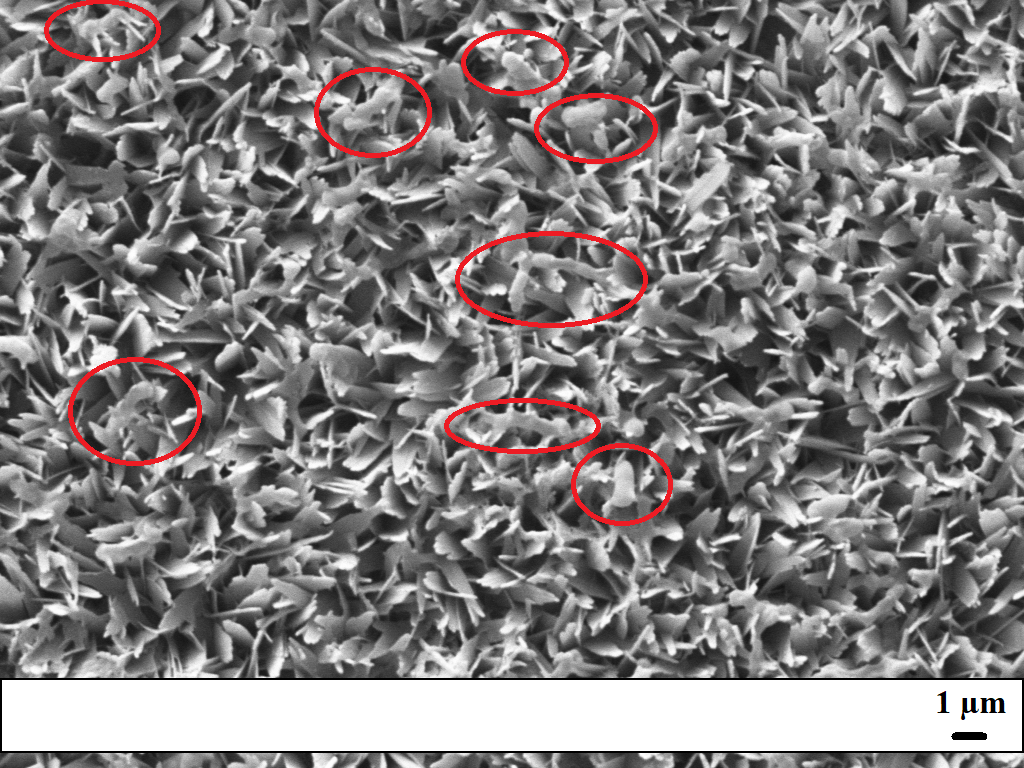


**Figure VI.** A high-magnification view of the HANb0 image, providing a clearer view of the *E. coli* bacteria over the surface.

**Supplementary Tables**

**Table I.** Chemical composition of SBF and PBS solutions.

| **SBF** | | **PBS** | |
| --- | --- | --- | --- |
| **Chemicals** | **Concentration (g/L)** | **Chemicals** | **Concentration (g/L)** |
| NaCl | 7.996 | NaCl | 8 |
| NaHCO_3_ | 0.350 | KCl | 0.2 |
| KCl | 0.224 | Na_2_H_2_PO_4_ | 1.42 |
| K_2_HPO_4_.3H_2_O | 0.228 | KH_2_PO_4_ | 0.27 |
| MgCl_2_.6H_2_O | 0.305 |  | |
| 1 M HCl | 40 mL/L |  | |
| CaCl_2_ | 0.278 |  | |
| Na_2_SO_4_ | 0.071 |  | |
| (CH_2_OH)_3_CNH_2_ | 6.057 |  | |

**Table II.** Quantitative results of the antibacterial rate obtained from plate-counting assay.

| **Sample type** | **Antibacterial Activity (%)** | |
| --- | --- | --- |
|  | ***E. coli*** | ***S. aureus*** |
| NiTi | 33.3 | 26.6 |
| HANb0 | 42.6 | 56.0 |
| HANb1 | 58.6 | 57.3 |
| HANb2 | 77.3 | 73.3 |
| HANb3 | 69.3 | 70.6 |

The antibacterial rate (K) of the samples was calculated using the following formula:

K =$\frac{X-Y}{X}$× 100

where, X and Y are the average number of colonies in the control and the average number of bacteria colonies in the studied samples, respectively. The initial concentration of *E. coli* and *S. aureus* bacteria was 7.5×10^3^ CFU/mL.
